# PET Waste Upcycling with Polysaccharides: Promising Alternative for Sustainability—Review

**DOI:** 10.1021/acsomega.5c06399

**Published:** 2025-12-18

**Authors:** Francisca P. Araujo, Denise B. França, Jessica G. Silva, Alisson Santana, Edson C. da Silva-Filho, Durcilene A. da Silva, Carlos A. P. Almeida, Josy A. Osajima, Edvani C. Muniz

**Affiliations:** † Interdisciplinary Laboratory for Advanced Materials (LIMAV), 67823 Federal University of Piauí, Avenida Universitária s/n, Teresina 64049-550, Piaui, Brazil; ‡ Federal University of Uberlândia, Graduate Program in Chemical Engineering, Avenida João Naves de Ávila 2121, Uberlândia 38408-100, Minas Gerais, Brazil; § Research Center on Biodiversity and Biotechnology (BIOTEC), Federal University of Parnaiba’s Delta, Avenida São Sebastião 2819, Parnaiba 64202-020, Piaui, Brazil; ∥ QUÍMICA, CEDETEG, 307046 Universidade Estadual do Centro-Oeste, Guarapuava 85040080, Paraná, Brazil; ⊥ Department of Chemistry, State University of Maringa, Avenida Colombo 5790, Maringá 87020-900, Paraná, Brazil

## Abstract

The accumulation of plastic waste is a challenge in today’s society owing to the several adverse effects on the environment caused by it. Among plastics, poly­(ethylene terephthalate) (PET) is a polymer that stands out as a significant source of pollution due to its extensive use in disposable packaging. Strategies involving PET upcycling can significantly contribute to the reduction of plastic waste. This perspective presents a recent trend in PET upcycling, focusing on the combination of PET waste with polysaccharides to obtain high-value-added materials. The influence of adding different types of polysaccharides and various preparation methods on the properties of recycled PET (rPET)-based membranes is thoroughly discussed. In addition, the relationship between the properties and applications of the rPET/polysaccharide membranes is addressed. In addition to demonstrating the potential and versatility of these materials, this review identifies challenges related to their industrialization, sustainability, and circularity, while also proposing future research directions for PET upcycling with polysaccharides.

## Graphical abstract

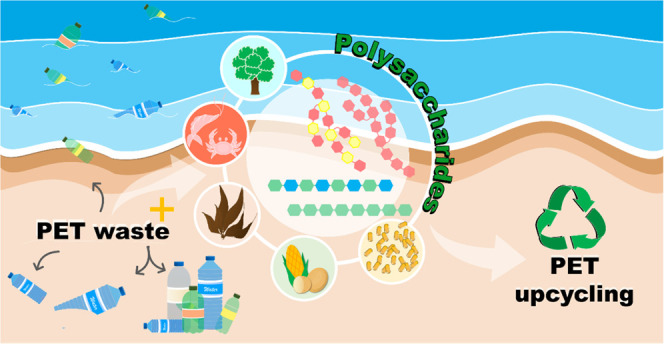

## 1 Introduction

The high production, consumption, and waste mismanagement of plastics have increased plastic pollution in the environment.^1–3^ According to the Plastic Overshoot Day report,^1^ there has been a consistent rise in global plastic waste generation (packaging, textile, and household), from around 206 million tons in 2021 to 220 million tons in 2024. On the other hand, it was estimated that around 31.5% of plastic waste (∼69.5 million tons) was mismanaged globally in 2024. The amount of plastic produced is expected to double by 2040, which will triple the volume of plastic pollution.^1^

One of the most commonly produced synthetic plastics is PET, with a global production rate of approximately 70 million tons/year.^4,5^ PET is a synthetic polymer of great economic importance in society due to its resistance, chemical properties, transparency, flexibility, and recyclability.^6^ PET is used in the packaging, textiles, and carpet sector and represents the main constituent of accumulated plastic waste in the environment.^7,8^ Due to its stability and resistance to degradation, PET discarded in the environment comprises around 12% of the global solid waste (in volume) and 8% by weight.^9,10^ In addition, the global PET market has grown in recent years. It is expected to continue following this trend in the coming years, leading to an increase in the volume of plastic waste as contaminants/pollutants.^11^

Plastic pollution adversely affects the environment, biota, and human health.^2,3,12^ Once plastics have entered the environment, they will inevitably break into microplastics^2,3,12^ that can be ingested by marine organisms, including those that are part of human nutrition.^13,14^ In addition, microplastic particles can also carry persistent organic pollutants and reach different living species, including humans.^15^ The concentration of these contaminants is expected to increase continuously unless practical actions related to plastic production and/or plastic waste management are taken.^16^ Removing these micropollutants from the environment is challenging, so preventive strategies for minimizing plastic pollution are desired.^12^

Recycling is a strategy to prevent plastic waste from reaching the environment.^17^ Mechanical and chemical recycling are the most popular processes for plastic recycling.^18–20^ The difference between these two methods is that in mechanical recycling, the plastic is ground and reprocessed by extrusion, while in chemical recycling, the macromolecule chain is transformed into its precursor monomers and can be used again in further polymerization.^18,21^ Currently, mechanical recycling is the best technology for PET recycling, with advantages such as low energy consumption and low cost.^18,19^ However, although PET is more widely recycled compared to other plastics, the current state of its recycling is far from ideal.^4,5^ Reported recycling rates for PET range from 19.5 to 49%.^4,22^ Factors contributing to these low recycling rates include (i) contamination of PET waste, which reduces the quality and usability of rPET; (ii) absence of efficient collection strategies and adequate infrastructure, especially in developing countries; and (iii) economic dominance of virgin PET.^4,21^ Thus, a significant amount of nonbiodegradable PET waste remains in landfills, rivers, and oceans.^10^

In this context, upcycling technologies have been proposed to increase the percentage of rPET.^23,24^ Upcycling technologies are advantageous because they convert plastic waste into high-value-added products.^24–26^ The increase in the added value of rPET products encourages the collection and recycling of this polymer and expands its application areas.^23,25^ Using plastic waste reduces the demand for virgin polymer and the production costs of new materials.^27,28^ The environmental impacts (global warming) for producing virgin synthetic polymers and plastic waste can be mitigated.^19,28^

This review addresses recent plastic upcycling strategies for obtaining materials that combine PET waste with polysaccharides. Polysaccharides are biodegradable, abundant, and renewable materials in nature.^29,30^ Among the polysaccharides, cellulose,^31,32^ chitosan,^23,33^ alginate,^27,34^ starch,^35^ and xanthan gum^36^ were used to obtain rPET-based materials. Polysaccharides can reinforce and/or functionalize the rPET matrix,^35,37,38^ and the combination between them results in materials with properties suitable for various applications, including water purification^23,32,34–36,39,40^ and mixture separation,^38,41^ as well as for the biocatalysis^31^ and biomedical^27,33^ fields.

Although some reviews have reported the influence of lignocellulosic fibers on the mechanical performance of rPET-based composite materials,^42–44^ this review focuses on membranes based on a combination of PET waste with polysaccharides, especially cellulose, chitosan, alginate, starch, and xanthan gum. The effect of each type of polysaccharide on the structure and properties of rPET-based membranes is highlighted and compared. In addition, the synthetic strategies used to modulate the properties of the rPET/polysaccharide materials are described. The relationship between the properties, applications, and performance of the rPET/polysaccharide membranes is also discussed. Therefore, this review describes the primary preparation methods, properties, and applications of rPET/polysaccharide membranes. Additionally, the study discusses the advantages and disadvantages of these materials in the context of plastic upcycling, addressing the opportunities and challenges for PET circularity. The objective is to provide a comprehensive overview of current scientific research on the combination of polysaccharides with PET waste to obtain high-value-added materials, as well as to identify the knowledge gaps. The findings can provide insights for future research and enhance the PET waste management efficiency.

## 2 General Characteristics of PET

PET, whose molecular formula of the repeat unit is (C_10_H_8_O_4_)_n_, is a polyester thermoplastic polymer of great industrial relevance. PET can be obtained by esterification or transesterification processes.^44,45^ In the esterification process, the bis­(hydroxyethyl) terephthalate (BHET) monomer is obtained from terephthalic acid (TPA) and ethylene glycol (EG). In transesterification, dimethyl terephthalate reacts with EG to form the BHET monomer.^46^ After esterification or transesterification processes, BHET undergoes a prepolymerization reaction to form oligomers, followed by polycondensation to form PET. The chemical representation of the structure of PET is shown in Figure 1. The polymer can be processed using different techniques to obtain plastic parts.^45^

**Figure 1:**
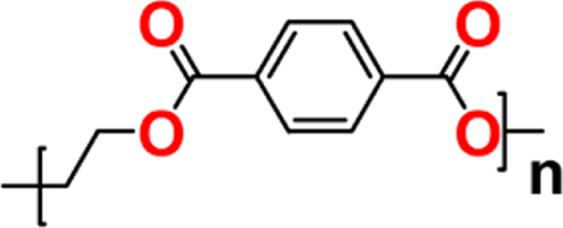
Chemical representation of PET structure (where ’n’ corresponds to the number of repeat units).

PET is a transparent polymer that has high stiffness, mechanical strength, and chemical resistance.^44^ Due to these properties, it is an excellent material for use in packaging, textiles, automotive components, construction materials, electronic components, and automotive manufacturing.^9,19,44^

## 3 Polysaccharides

Polysaccharides are natural compounds formed by the union of many monosaccharide units linked by glycosidic bonds and are considered the most common biopolymers in the world.^47^ These macromolecules can be found in plants, animals, algae, and microorganisms and are classified according to their origin, function, chemical composition, and presence (or not) of charges (cationic or anionic).^48–50^ In addition, the chain’s polysaccharide can be linear or branched.^51^

Polysaccharides have interesting properties such as biodegradability, biocompatibility, high natural availability, nontoxicity, and hydrophilicity. They are in great demand for obtaining ecological and sustainable materials.^29,30,52–54^ Additionally, they play important roles in various fields, such as the removal of environmental contaminants,^55–57^ tissue repair,^58,59^ agriculture,^29,60,61^ packaging materials,^62,63^ and others.

Recently, studies have demonstrated the potential of polysaccharides in PET upcycling processes. These processes are related to the design and synthesis of high-value-added functional materials based on combining polysaccharides with waste PET.^34,40,64,65^ Polysaccharides that were combined with waste PET to obtain different types of materials include cellulose,^31,32^ chitosan,^23,33^ alginate,^27,34^ starch,^35^ and xanthan gum.^36^ The structures of these polysaccharides are shown in Figure 2.

**Figure 2:**
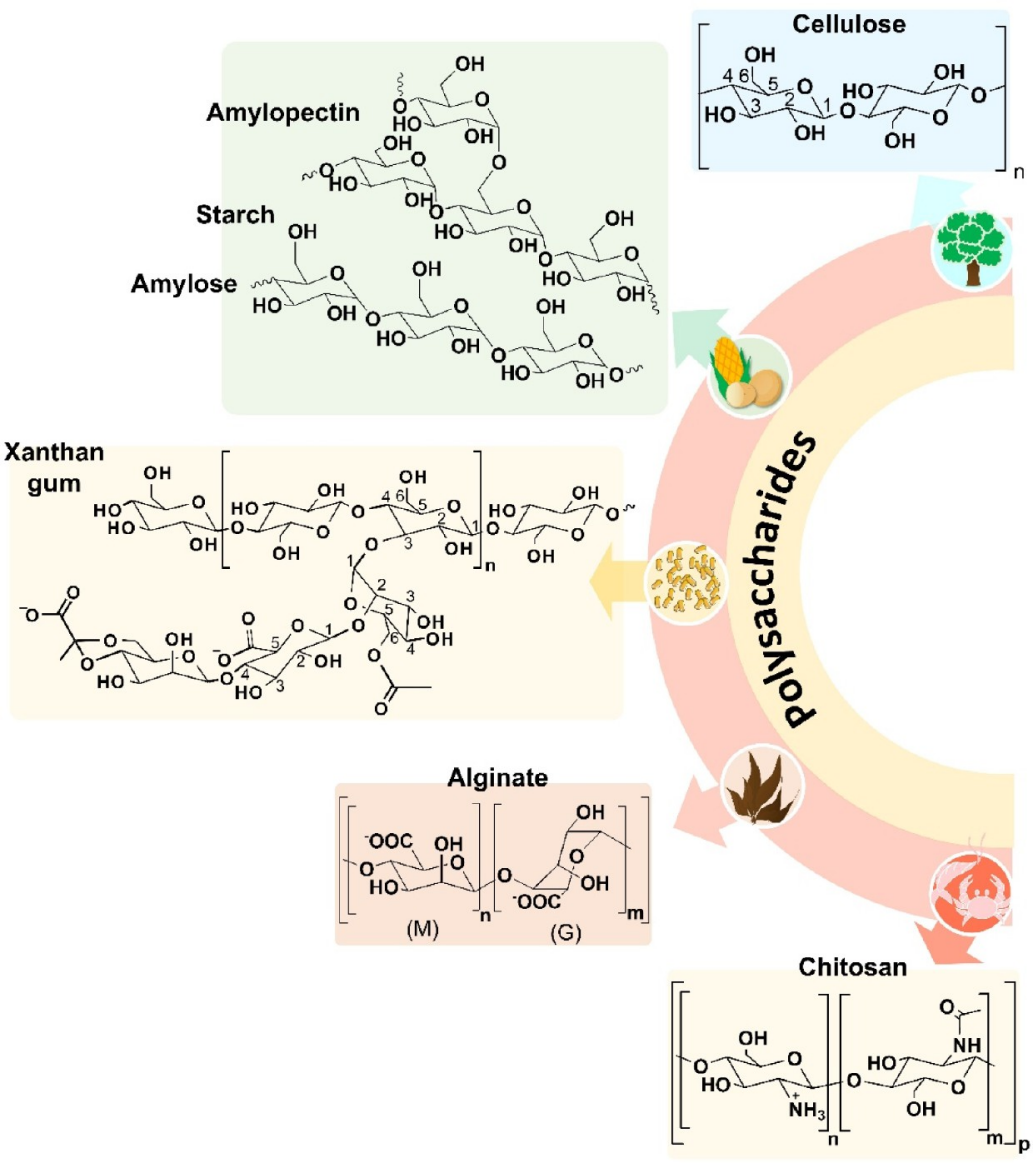
Structure of the polysaccharides used for the preparation of materials based on PET waste.

### 3.1 Cellulose

Cellulose is one of the most abundant materials on earth, found or produced by various plants, tunicates, bacteria, and other living organisms.^66,67^ This polysaccharide is produced at approximately 10^11^–10^12^ tons per year.^67^ Cellulose is an unbranched neutral homopolysaccharide composed of d-glucose units joined by β-1.4 glycosidic bonds (Figure 2). It can be processed into several forms, such as microfibrils, microcrystals, nanofibrils (CNFs), and nanocrystals (CNCs).^68^ This polysaccharide has high mechanical strength, flexibility, thermal stability, high availability of hydroxyl groups, and hydrophilicity.^66,67,69,70^ The mechanical properties of cellulose depend on the source, morphological form, and extraction procedure. Elastic modulus, tensile strength, and elongation to rupture values in the range of 5–220 GPa, 300–7700 MPa, and 1–22%, respectively, were reported for cellulose.^69^ However, the highest mechanical properties are reported for nanocellulose, like CNCs and CNFs.^69^ Thus, nanocellulose is mainly applied as reinforcement in polymer composites,^68,69^ including rPET-based materials.^37^

### 3.2 Chitosan

Chitosan is the *N*-deacetylation product of chitin, which is also a polysaccharide abundant in nature and biosynthesized by fungi, plankton, insects, and crustaceans.^71,72^ About 10^10^–10^12^ tons/year of chitin are naturally produced.^72^ The chitosan structure comprises d-glucosamine and *N*-acetyl-d-glucosamine joined by β-1.4 glycosidic bonds (Figure 2).^72^ Due to the presence of –NH_2_ groups in its structure, chitosan is one of the rare polycationic polymers (p*K*_a_ ∼ 6.3) in nature, which distinguishes it from other polysaccharides.^72,73^ In addition, chitosan has antibacterial properties.^74^ However, chitosan presents some disadvantages that restrict its application in specific fields, such as its solubility primarily in aqueous solutions of diluted acids and its poor thermal and mechanical properties.^72,75^ Combining polysaccharides with rPET is one strategy to overcome these limitations.^33^

### 3.3 Alginate

Alginate is a linear polyanionic polysaccharide found in the cell wall and intercellular mucilage of different species of brown algae, as well as in some bacteria, such as Pseudomonas and nitrogen-fixing bacteria, mainly in the form of sodium salts.^76,77^ This macromolecule is composed of α-l-glucuronic acid (G) and β-d-mannuronic acid (M) monomeric units, which are irregularly linked by β-1,4-glycosidic bonds (Figure 2) to form GG blocks, MM blocks, and alternating M and G units within the larger polymer.^76,78,79^ The distribution and proportion of these three blocks in alginate depend on factors such as the source, geographical origin, degree of maturity, and harvesting time.^78^

### 3.4 Starch

Starch is also an abundant polysaccharide, which is mainly derived from the roots, stalks, and seeds of common crops such as corn and potato.^63,80^ It comprises two different polysaccharide structures, amylose and amylopectin (Figure 2). Amylose is a linear polysaccharide in which α-1,4-glycosidic linkages link d-glucose units. In contrast, amylopectin is a highly branched polymer composed of short chains of α-(1,4)-linked d-glucose units with branches formed by α-(1,6) linkages at the branch positions.^63,80,81^ Amylopectin is the major component of starch (70–80%).^81^

### 3.5 Xanthan Gum

Xanthan gum (XA) is a heteropolysaccharide produced by *Xanthomonas campestris* and other *Xanthomonas* spp. during aerobic fermentation.^82,83^ Its primary structure consists of repeated pentasaccharide units formed by d-glucose, d-mannose, and d-glucuronic acid, with a molar ratio of 2.0:2.0:1.0.^83^ The unbranched chain structure of XA consists of d-glucose residues linked via β-1–4 glycosidic linkage, which is similar to that of the cellulose chain.^82,84^ The polymer’s branching structure results from glucuronic acid residues connected to specific mannose units. β-1.4 glycosidic bonds join together the d-glucose and d-mannose units, whereas the d-glucuronic acid residues are linked to the d-mannose units via β-1.2 glycosidic bonds.^85^ The structure of XA is presented in Figure 2.

## 4 PET Upcycling: Materials Based on PET Waste and Polysaccharides

A more promising approach to recycle waste PET is to use it to obtain high-value-added functional materials.^24^ The materials obtained from the upcycling of PET with polysaccharides include mats,^37,64,65^ membranes,^23,32,35,36,38–41^ filaments,^86^ and fibers.^31,33,87^ The PET sources, polysaccharides used, preparation methods, and applications of rPET/polysaccharide materials are listed in Table 1.

**Table 1:** PET Waste Associated with Polysaccharides for Membrane, Filaments, Fibers, and Mats Fabrication and Application

material	PET source	polysaccharide	method	application	reference
mats	rPET (melt flow index of 36.4 g (10 min)^−1^	cellulose from sisal	electrospinning (static collector)	-	^64^
			electrospinning (rotary collector)	-	^65^
		cellulose nanocrystals	electrospinning (static and rotary collectors)	-	^37^
membrane	waste Coca-Cola PET bottles	cellulose from waste qualitative filter paper	electrospinning	separation of surfactant-stabilized water-in-oil and oil-in-water emulsions	^39^
	drink from PET bottles marked with the recyclability code – 1^a^	cellulose from waste papers	nonsolvent-induced phase separation technique	separation of oil–water emulsions	^32^
nanofibrous membrane	wastewater PET bottles with recyclability code – 1^a^	cellulose acetate	electrospinning	carriers to porcine pancreatic lipase	^31^
filament	unused PET drinking water bottles	cellulose from the cotton fibers	extrusion	3D printing feedstock materials	^86^
membrane	waste PET bottles	chitosan	electrospinning	removal of hexavalent chromium	^40^
	recycled PET pellets from postconsumer PET water bottles	chitosan	electrospinning	oil–water separation	^23^
	waste PET water bottles	chitosan	interfacial polymerization using 2,5-furandicarboxaldehyde as a cross-linker for CS, and eucalyptol was used as an organic phase	organic solvent nanofiltration	^38^
	PET sheets obtained from reverse osmosis membranes that were discarded from the industry	chitosan	-	separation of ethanol/water and limonene/linalool synthetic mixtures	^41^
nanofibrous membrane	waste PET bottles	chitosan	electrospinning	obtaining single-use nonwoven fabric or biodegradable tissues for hygiene care	^33^
	PET bottles	chitosan	electrospinning	biomedical applications	^88^
filaments	waste PET bottles	chitosan	extrusion	-	^89^
fiber composites	PET waste textile fiber	alginate (fibers with 8–12 wt % CaO, 1.67 dtex × 38 mm)	opening-combing-needle punching with hot pressing	fire-proof valuable material in construction and building	^87^
porous material (membrane)	waste PET water bottles	alginate	nonsolvent-induced phase separation	dye removal	^34^
membrane	-	alginate	electrospinning	anti-infective therapy (wound dressings)	^27^
biomimetic membrane	waste PET drink bottles	potato starch	electrospinning and vacuum filtration	water-in-oil emulsion separation	^35^
membrane	waste PET bottles	xanthan gum (XA)		removal of diltiazem from aqueous solution by nanofiltration	^36^

a PET bottles marked with code-1 indicates that the material used to make the bottle is only PET without any blending with other polymers.

The hydroxyl groups of polysaccharides can interact with carbonyl groups from the PET chains via hydrogen bonds.^32,35,64,65^ Additionally, interaction can be improved by modifying the rPET matrix^40^ or polysaccharide,^39,86^ as well as by using compatibilizers.^32,37^ The regulation of compatibility between natural and synthetic polymers is an important aspect in determining the properties of the material. Good interaction between the polymers is highly desirable because poor adhesion may lead to unsatisfactory mechanical properties of the resulting material.^37^ For instance, rPET/polysaccharide materials with good interfacial compatibility presented improved mechanical properties.^37,39^ Standard methods used to estimate polymer–polymer compatibility include viscometry, Fourier transform infrared (FTIR) spectroscopy, and, especially, differential scanning calorimetry for determining glass transition temperatures (*T*_g_) and, consequently, the number of phases that coexist in a polymer blend.^89,90^

To develop materials based on blends of rPET and polysaccharides, the components can be combined in the molten state (melt mixing) or dissolved in the same solvent. Typical solvents include trifluoroacetic acid (TFA)^32,33,37,64,65^ or a TFA/dichloromethane binary system.^34,39^ However, rPET/polysaccharide materials can also be obtained by functionalizing an rPET matrix with the polysaccharide.^27,35,40,88^ In this case, the rPET matrix is used as a support for the polysaccharide. Nevertheless, the polysaccharide was also used as a support matrix for rPET.^39^

Most polysaccharides do not have satisfactory mechanical strength,^63,87,91^ and their association with synthetic polymers can be a strategy to provide a material with improved mechanical properties.^39,41^ In addition, polysaccharides introduce new properties and functionalities to the material prepared from waste PET.^23,32,36,37^ These properties depend on the polysaccharides’ extraction/purification/preparation methods, type, and content. Adding polysaccharides is a strategy that can also be used to improve the mechanical properties^32,37^ of the rPET matrix, as well as its hydrophilicity.^23,36,64^ Due to their chemical nature, materials prepared from PET tend to be highly hydrophobic, which is a disadvantage in some applications. For instance, the hydrophilicity of PET-based materials is important for their application in water purification processes.^23,32,34–36,39,40^ Functional groups of polysaccharides can also interact with species, such as dyes, drugs, and metal ions, for which PET has weak or no affinity.^34,36,40^ Thus, rPET/polysaccharide materials can be used in the treatment of water contaminated with these pollutants. Furthermore, the ability to interact with metal ions can be used to prepare materials with antibacterial properties for use in the biomedical field.^27^ Therefore, polysaccharides contribute to expanding the application field of rPET. Indeed, materials based on rPET and polysaccharides have properties that are suitable for applications in a variety of areas. The potential of these materials was reported in water purification^23,32,34–36,39,40^ and mixture separation^38,41^ processes, biocatalysis,^31^ and the biomedical field^27,33^ (Table 1). The application field depends on the properties and type of material produced.

Cellulose and chitosan were the polysaccharides mostly used to prepare materials based on waste PET. At the same time, postconsumer bottles are the primary source of PET, which are previously cleaned (with detergent, water, ethanol, and others), dried, and cut into flakes.^31,32,35,36,89^ Depending on the impurities present, other treatments may be carried out before recycling the PET.^21^ This process is imperative to mitigate adverse effects on the quality and purity of rPET and is also necessary in other PET recycling methods (mechanical, chemical, and biological recycling).^21^ The processed PET flakes are then used to prepare rPET/polysaccharide materials without the need for prior mechanical (extrusion) or chemical recycling. However, mechanically recycled PET has also been used in some studies.^37,64,65^

Using PET bottles or rPET to produce new materials minimizes the demand for virgin PET. It contributes to the removal of PET-based contaminants from the environment, conservation of raw petrochemical products and energy, and reduction of greenhouse gas emissions.^21,42,44,92–94^ Thus, PET waste conversion to value-added products such as rPET/polysaccharide membranes can be considered as a sustainable approach.

### 4.1 rPET/Polysaccharides Membranes or Mats

Membranes and mats were the main materials obtained from the combination of PET waste and polysaccharides.^23,32,35,36,38,39,41^ Membranes are selective and semipermeable materials used in the food industry, pharmaceuticals, fuel cells, and gas separation, but they mainly stand out in water purification.^95,96^ Cellulose, chitosan, starch, and xanthan gum were used to produce the rPET-based membranes. This section describes the preparation and properties of rPET/polysaccharide membranes and mats, highlighting the main effects caused by each polysaccharide (cellulose, chitosan, alginate, starch, and xanthan gum).

#### 4.1.1 Membranes or Mats Based on rPET and Cellulose

Cellulose was used to improve the hydrophilicity, porosity, and mechanical properties of rPET-based membranes or mats.^32,37,39,64,65^ CNCs,^37^ CNFs,^32^ cellulose from sisal pulp,^64^ and cellulose from qualitative filter paper^39^ were investigated for this proposal. Methods such as electrospinning^37,39,64,65^ and the nonsolvent-induced phase separation technique (NIPS)^32^ were used to obtain these materials. Most rPET/cellulose membranes or mats were obtained from the blend of both polymers.^32,37,64^ The properties of membranes depend on the synthesis method, experimental preparation conditions,^37,64,65^ compatibility or interaction between the components,^32,37,39^ and cellulose content.^32,39^

Interaction of cellulose with rPET resulted in materials with improved properties compared to the individual components.^32,64,65^ A mat obtained from a blend of rPET and cellulose (from sisal pulp) by electrospinning, using trifluoroacetic acid (TFA) as the solvent, presented higher average fiber diameter, glass transition temperature (*T*_g_), and tensile strength compared to the neat rPET mat (Table 2).^64^ These results indicated stronger interactions at the molecular level between the PET chains and cellulose. In addition, the viscosity of the solution prepared from rPET and cellulose (2484 ± 13 cP) was higher than that of individual rPET (55.2 ± 0.5 cP) or cellulose (441 ± 6 cP) solutions due to the interaction between them in the solution phase.^64^ According to the contact angle (CA) results, cellulose introduced a hydrophilic character to the rPET mat due to the hydrophilic hydroxyl groups on its surface. In contrast, the neat rPET mat was hydrophobic (Table 2). Therefore, cellulose can be used to tune various properties of rPET-based materials.

**Table 2:** Some Properties of Electrospun rPET/Cellulose Mats (Obtained at a Dissolution Time of 72 h)^a^

mat	PET (g dL^–1^)	cellulose (g dL^–1^)	Ra^b^ (nm)	*T*_g_ (°C)	tensile strength (MPa)	CA^c^ (°)
rPET	15.0	0	242 ± 59	92.5 ± 0.1	1.8 ± 0.2	123.8 ± 6.8
rPET/cellulose	15.0	1.3	366 ± 139	111.3 ± 0.7	9.5 ± 0.6	54.4 ± 4.6

a Data obtained from Santos et al.^64^

b Average fiber diameter.

c Advancing (maximum) CA.

The interaction between cellulose and rPET can be improved by using compatibilizers.^32,37^ This strategy was used to obtain rPET/cellulose mats with improved properties.^37^ For example, the incorporation of castor oil (CO) as a compatibilizer between rPET and cellulose nanocrystals (CNCs) increased the hydrophilicity, storage modulus (Figure 3a), tensile strength (Figure 3b), and elastic modulus (Figure 3c) values of an electrospun rPET/cellulose mat obtained using a stationary fiber collector.^37^ CO is mainly composed of the triglyceride of ricinoleic acid, which has chemical structure moieties with hydrophobic (hydrocarbon chain) and hydrophilic (carbonyl and hydroxyl groups) characteristics that can interact attractively with PET and cellulose. In addition, the presence of CNCs increased the *T*_g_ values of rPET/CNC (109.6 °C) and rPET/CO/CNC (108 °C) compared to those of rPET (92 °C) and rPET/CO (91.3 °C), which confirms the interaction between the polymers.^37^

**Figure 3:**
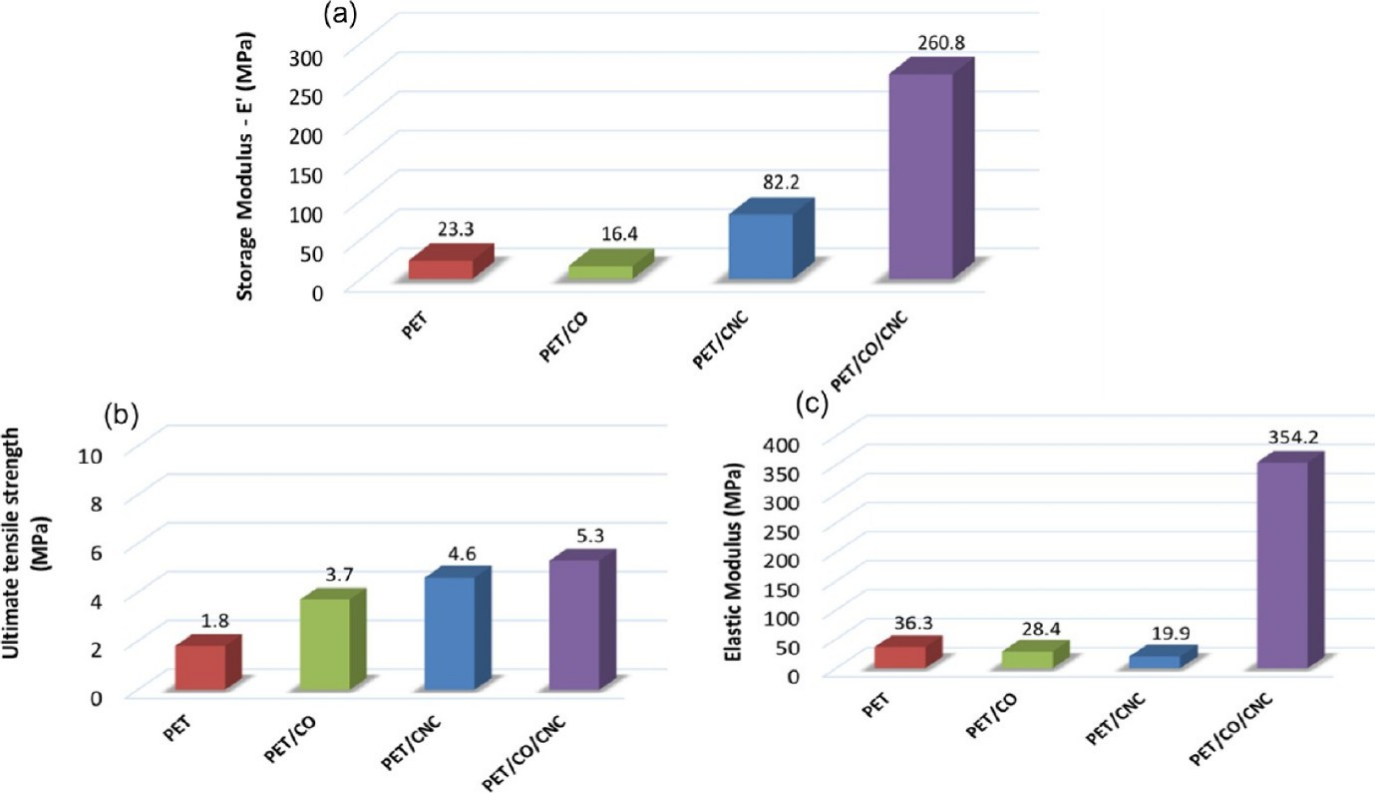
Different properties of rPET (PET), rPET/castor oil (PET/CO), rPET/cellulose nanocrystals (PET/CNC), and rPET/castor oil/cellulose nanocrystals (PET/CO/CNC) mats: (a) storage modulus (at 30 °C); (b) ultimate tensile strength; and (c) elastic modulus. Adapted with permission from Santos et al.^37^ Copyright 2025 Elsevier.

Modifications of cellulose were also carried out to make the polysaccharide more compatible with an rPET matrix.^39,86^ The chosen compatibility strategy depends on the type of material prepared. For example, polydopamine (PDA) acts as a structural adhesive between the layers of cellulose and rPET of a Janus membrane, preventing its delamination during the mechanical stretching process.^39^ The membrane was prepared by electrospinning rPET solution onto a modified PDA cellulose superhydrophilic membrane to form a hydrophobic PET layer. Using this strategy, it was possible to obtain an rPET/PDA-cellulose Janus membrane with asymmetric wetting properties suitable for the separation of oil-in-water and water-in-oil emulsions. In addition, the tensile stress (12.0 MPa) of the rPET/PDA-cellulose Janus membrane was 83% higher than that of the PDA-cellulose membrane (8.8 MPa), which shows the importance of rPET in the mechanical properties of the material.

Another study verified that poly­(ethylene glycol) (PEG-400) was crucial in forming blend membranes of rPET and CNF prepared by the NIPS technique using distilled water as the nonsolvent. PEG-400 acted as a plasticizer and a compatibilizer inside the polymeric matrix.^32^ Furthermore, the CNF content (1.0, 1.5, 2.0, and 2.5% wt/vol) inside the PET matrix greatly influenced the surface properties of the membrane. Scanning electron microscopy (SEM) results (Figure 4a-b) showed that CNFs in the membranes had a significant role in pore formation. A decrease in CA values and increased water absorption and porosity percentages was observed up to the cellulose concentration of 2.0% (Figure 4c–e), and above 2% of cellulose, water absorption and porosity percentages decreased, probably due to nanofiber agglomeration of CNFs over the pores after the optimum concentration. However, a decrease in tensile strength value was observed after the incorporation of CNF into the PET matrix. The membrane fabricated using only PET had a tensile strength value of 2.33 MPa. After incorporating CNF at 1.0, 1.5, 2.0, and 2.5 wt %/vol, the tensile strength values were 1.19, 0.95, 0.86, and 0.76 MPa, respectively. Despite the reduction, the tensile strength values of rPET/cellulose membranes obtained by the NIPS were still sufficient for their application in oil–water separation.^32^ This result, related to the highly porous structure of the rPET/cellulose membrane obtained by the NIPS, shows a relationship between the properties of rPET/polysaccharide membranes and the preparation method.^32^

**Figure 4:**
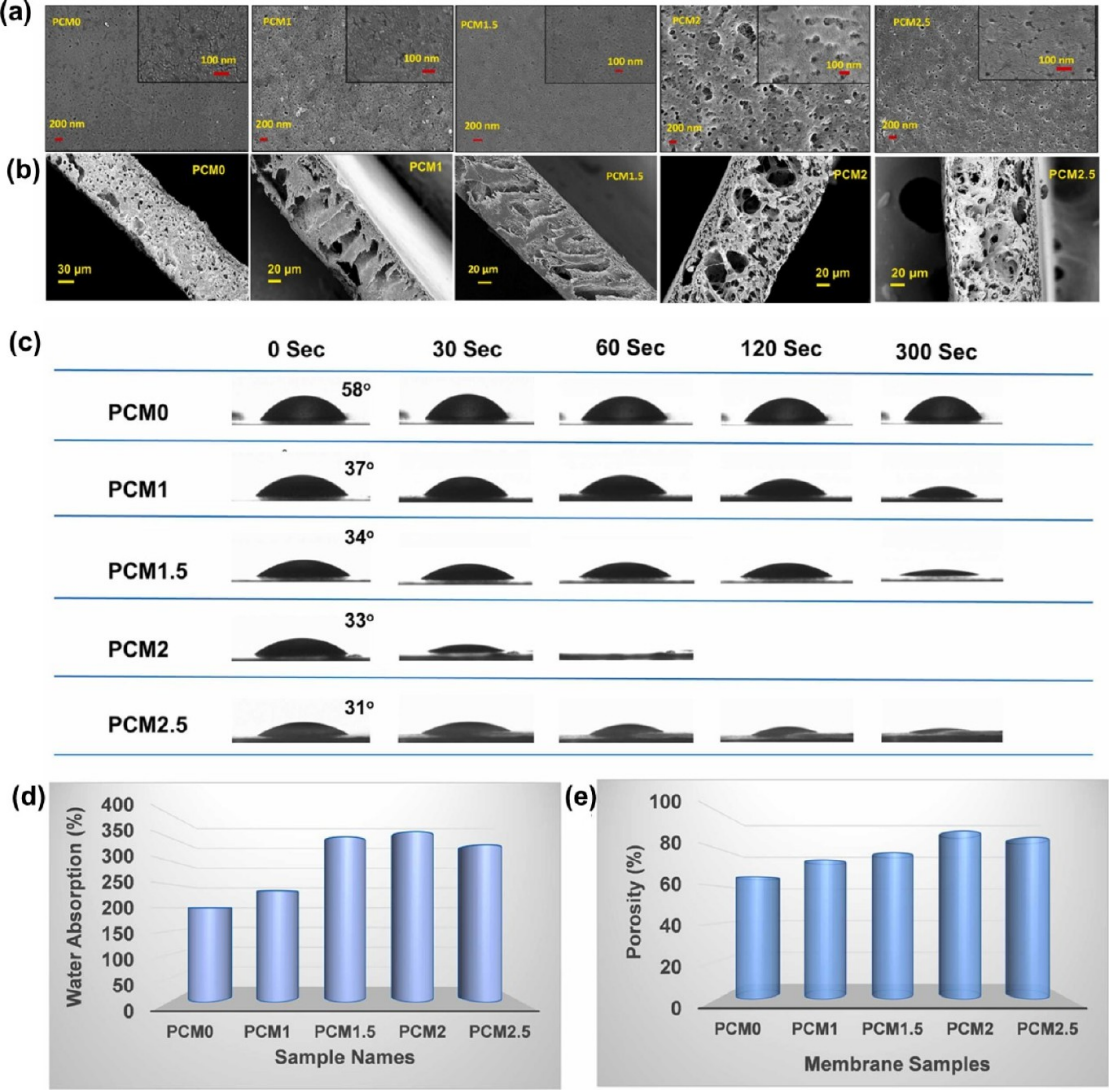
(a) SEM images of the surfaces; (b) cross-section of different PET and PET/cellulose membranes; (c) CA analysis; (d) water absorption analysis of the membranes; and (e) porosity of the designed membrane. PCM0, PCM1, PCM1.5, PCM1.5, PCM2, and PCM2.5 refer to membranes that contain. 0%, 1%, 1.5%, 2%, and 2.5%, respectively. Adapted with permission from Bhuyan et al.^32^ Copyright 2025 Elsevier.

The low tensile strength values of rPET/CNF membranes obtained by NIPS may hinder the reusability and durability of the membrane, thereby reducing its useful life. Indeed, regeneration and reuse of membranes tend to cause a decrease in their mechanical properties.^35^ Although the durability of the rPET membrane with 2% cellulose prepared by the NIPS was verified through the crossflow permeation experiment continuously up to 7 days, its regeneration and reusability were not investigated.^32^ In contrast, the membranes obtained by electrospinning had improved mechanical properties (tensile strength, storage modulus, ultimate tensile strength, impact resistance, impact strength, etc.) by the addition of cellulose (see Table 2 and Figure 3).^37,39,64^ Therefore, when the preparation method is chosen, the application of the material must be considered.

#### 4.1.2 Membranes Based on rPET and Chitosan

Membranes were the main materials produced by combining postconsumed PET and chitosan, whose preparation methods include electrospinning and interfacial polymerization (IP), using different synthesis strategies.^23,38,40,41^ Most rPET/chitosan membranes were obtained using rPET as a support.^38,40,41^ The preparation of these membranes generally involves two steps: in step I occurs the preparation of the rPET support and in step II occurs the incorporation of the polysaccharide into the rPET support.^23,38,41^ Depending on the application, a third step of cross-linking the chitosan may also be necessary to increase its stability in acidic media and decrease its degree of swelling in water.^38,41^ However, membranes based on the blends of rPET and chitosan were also obtained.^23,33^

The synthetic polymer is responsible for providing the appropriate mechanical properties and stability for the application of rPET/chitosan-based materials.^38,40,41^ The resistance in aqueous media and thermal stability of rPET/chitosan hybrid nanofibrous membrane obtained by electrospinning were greater than those of the chitosan nanofibrous membrane.^33^ Furthermore, improved mechanical properties (Young’s modulus, tensile strength, and elongation at break) and a lower degree of water swelling were observed for a rPET-supported chitosan membrane compared to the nonsupported chitosan membrane.^41^

On the other hand, chitosan may affect the water wettability of rPET-based materials.^23,89^ In contrast to the hydrophobic neat rPET materials, the nanofibrous membrane fabricated from the blend of rPET and chitosan by electrospinning exhibited superhydrophilic behavior.^23^ These membranes were obtained by electrospinning of homogeneous polymeric solutions prepared by dissolving chitosan (1.0, 1.5, 2.0, and 2.5%) and pellets from postconsumer rPET water bottles in TFA. Fibers with a more homogeneous shape and larger average diameter were obtained by adding chitosan to the polymeric solution, according to SEM results (Figure 5). In addition, fiber diameter and roughness of the membranes were influenced by chitosan content, reaching maximum values for 2% of the polysaccharide. The addition of chitosan did not affect the superoleophilicity of the rPET membranes.^23^

**Figure 5:**
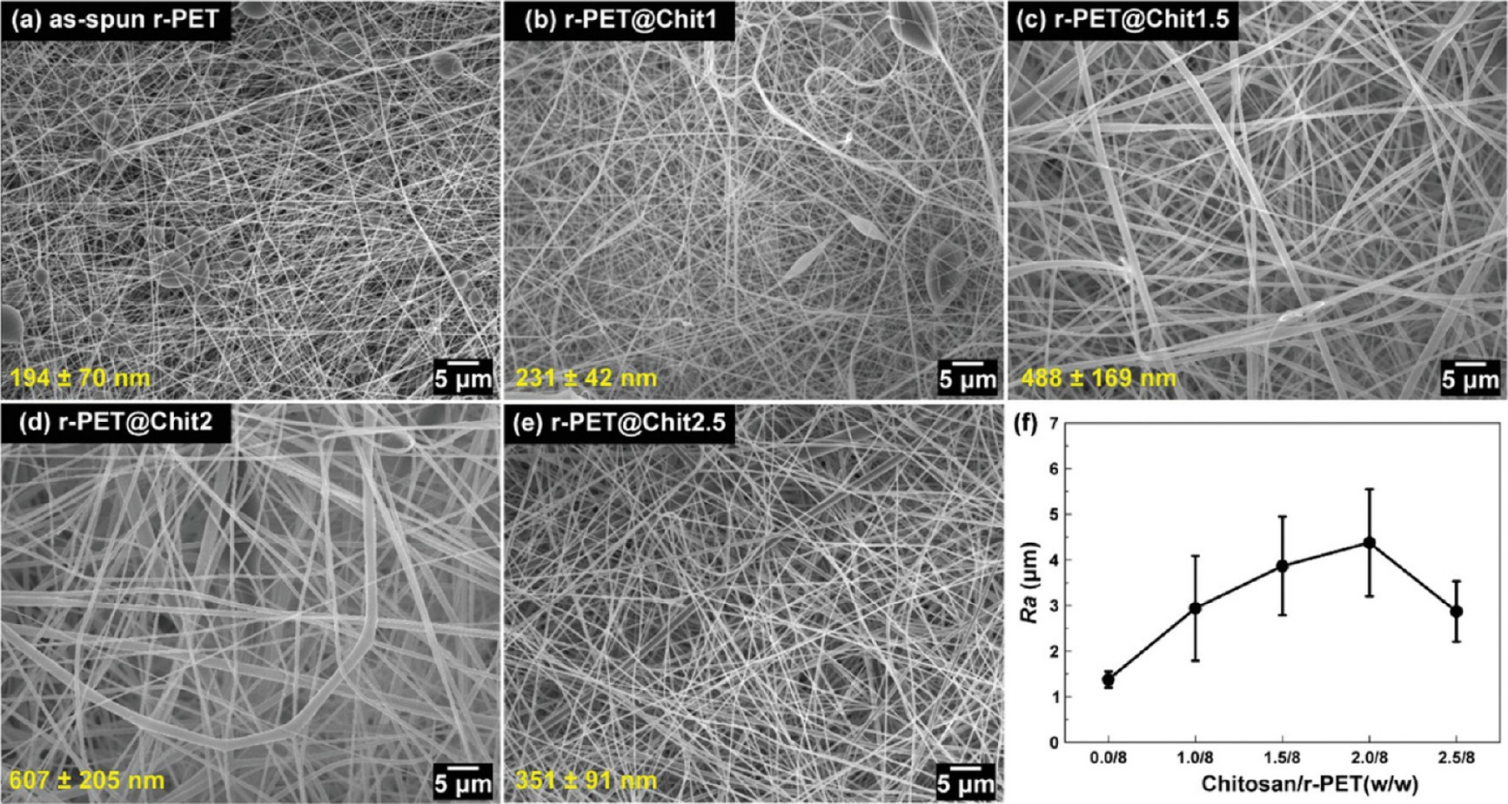
SEM images of: (a) rPET; (b) rPET@Chitosan1; (c) rPET@Chitosan1.5; (d) rPET@Chitosan2; (e) rPET@Chitosan2.5; and (f) plot of average fiber diameter for the membranes as a function of concentration of chitosan/rPET (w/w). Reprinted with permission from Baggio et al.^23^ Copyright 2025 John Wiley and Sons.

Thin-film composite (TFC) membranes were also prepared via IP reaction of chitosan (in aqueous acetic acid solution 2% v/v) and 2,5-furandicarboxaldehyde (FDA, in eucalyptol) on top of the recycled porous PET support, using 1,1,3,3-tetramethylguanidine (TMG) as a catalyst.^38^ FDA was used as a cross-linking agent. The incorporation of chitosan into the rPET support and imine bond formation by the Schiff base reaction of the amine and aldehyde moieties of the chitosan and FDA were verified by solid-state ^13^C NMR, FTIR, and XPS analysis. SEM images showed that the chitosan-based layer fully covered the porous rPET supports (Figure 6a–d). The thickness of the chitosan thin layer on the rPET support was found to be approximately 30 nm using the TEM image (Figure 6f). The water CA of the chitosan-based TFC membrane increased from 54.3 ± 1.7 to 67.4 ± 1.5° as a result of the increase in the FDA concentration. In contrast, the change in the chitosan concentration did not significantly influence the TFC membranes’ hydrophilicity.^38^

**Figure 6:**
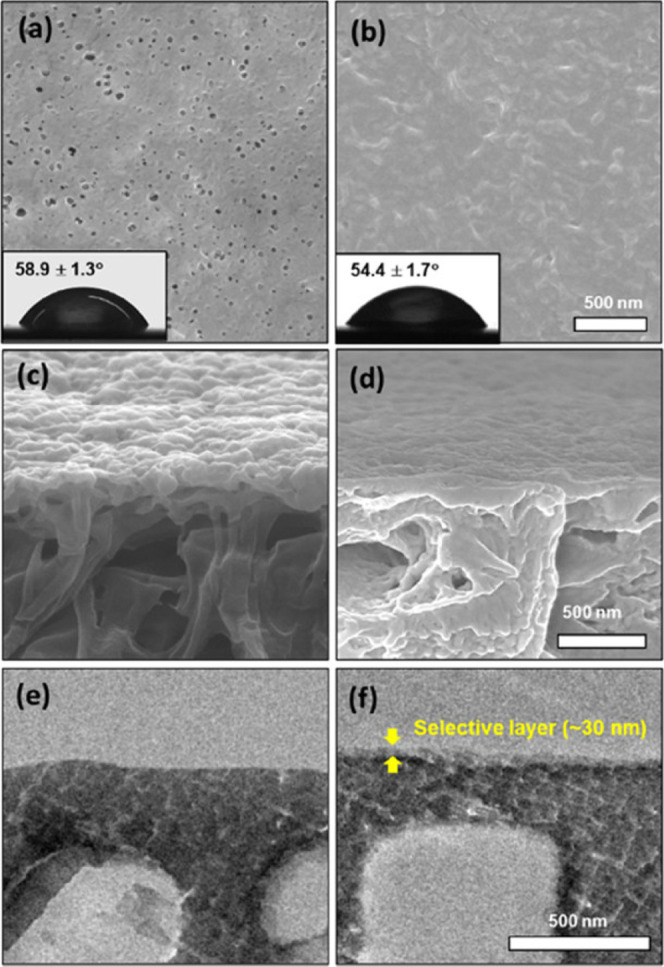
(a,b) Top surface SEM images; (c,d) cross-sectional SEM images; (e,f) cross-sectional TEM images of (a,c,e) the rPET support; and (b d,f) the chitosan-based TFC membranes. Insets are the digital camera images of the water CA. Membranes were fabricated at 0.5 mmol/v % chitosan and 3 mmol/v % FDA concentrations. Adapted with permission from Park et al.^38^ Copyright 2021 American Chemical Society.

To prepare another rPET/chitosan membrane, an activation step of the rPET support with cold plasma was performed to provide binding sites for chitosan onto the rPET surface.^40^ The incorporation of chitosan was verified by FTIR results, which showed that the spectrum of functionalized PET nanofibers with chitosan exhibited characteristic bands of polysaccharide at 3428 cm^–1^, 1570 cm^–1^, and 1298 cm^–1^.

Unlike cellulose, chitosan was mainly used to functionalize the rPET matrix, while its effect on the mechanical properties of the rPET-based material has been little studied.^38,40,41^ When inserted into the rPET matrix, the chitosan functional groups (−NH_2_ and –OH) play an important role in the reactivity and applicability of the prepared materials. However, the functionality of these materials has not yet been explored in the context of PET recycling. Additionally, the properties of the rPET/chitosan materials were influenced by the synthesis methods. For example, membranes with wettability higher or lower than that of the corresponding rPET material were obtained. Therefore, some properties of rPET/chitosan materials can also be adjusted by the proper choice of the synthesis method.

#### 4.1.3 Membranes Based on rPET and Alginate, Xanthan Gum, and Starch

Membranes based on rPET and alginate,^27,34^ XA,^36^ and starch^35^ were also obtained. The effect of the sodium alginate (SA) addition (9, 17, 23, 28, and 33 wt %) on the porous structure of composite membranes based on rPET prepared by the NIPS method was studied.^34^ The membranes were fabricated from a blend of rPET and SA. SEM images (Figure 7) showed that the increase in SA content hindered pore formation on the surface of the composite, and the pore size gradually decreased. In addition, the cross-sectional SEM images showed that a high alginate content reduced the homogeneity and density of the pores inside the composite but promoted the formation of hollow channels; the higher the alginate content was, the larger the channel size.^34^ In addition, the peaks at 3500–3300 cm^–1^ (O–H) and 1623 cm^–1^ (C=O) observed in the FTIR spectra obtained for the SA@PET composites proved that SA was successfully blended into the PET matrix.^34^ Unlike cellulose, sodium alginate did not improve the porosity of the membrane obtained with rPET prepared by the NIPS method.^32^ The influence of the polysaccharide on the hydrophilicity and mechanical properties of the materials was not investigated.

**Figure 7:**
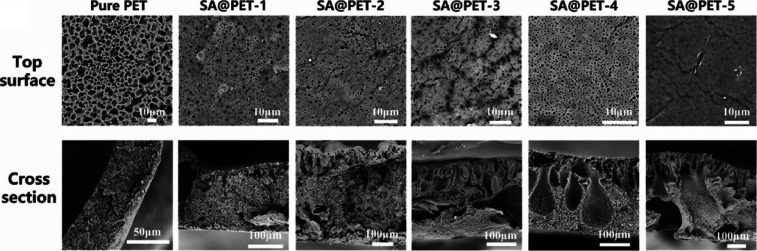
Top surface and cross-sectional morphology of the sodium alginate@PET (SA@PET) composite membranes prepared with different sodium alginate (SA) mass fractions. Adapted with permission from Yu et al.^34^ Copyright 2025 Elsevier.

XA (0.25, 0.50, 0.75, and 1.0 wt %) was incorporated into the membrane manufacturing from waste PET bottles as a hydrophilic additive.^36^ During membrane fabrication, the effect of the nonsolvent on membrane properties was investigated by using water or methanol as the nonsolvent. The incorporation of XA led to the increased porosity and thickness of the resultant blend membranes, which were higher for those prepared using methanol as a nonsolvent (Figure 8). Due to the porous structure, all rPET/XA membranes have lower mechanical properties than the rPET membrane. Furthermore, membranes prepared in methanol exhibited higher hydrophilicity due to the higher amount of XA remaining in this group of membranes. Thus, XA improved the porosity and hydrophilicity of rPET membranes but reduced their mechanical properties. Similar results were reported for the rPET/cellulose membrane obtained by the NIPS method.^32^

**Figure 8:**
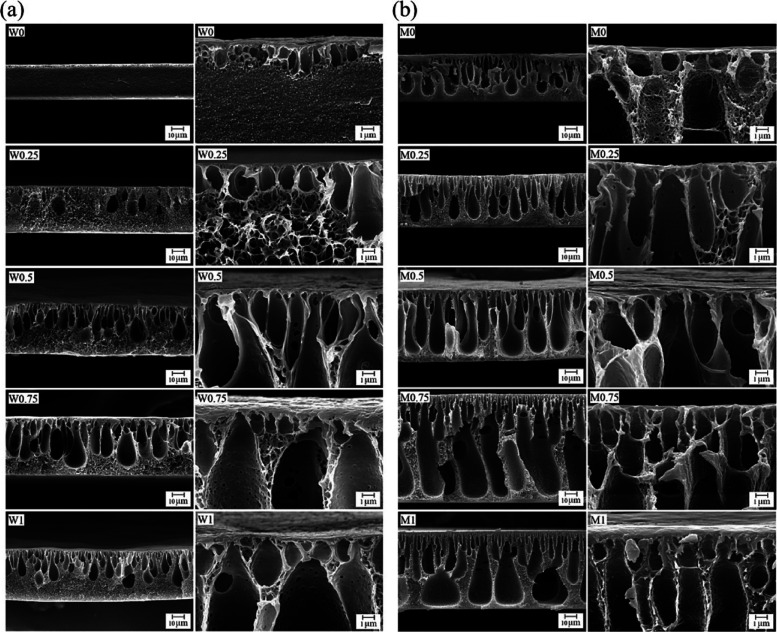
Cross-sectional SEM images of PET membranes prepared with different concentrations of xanthan gum in the nonsolvent bath of (a) water and (b) methanol (left: 2500× and right: 25,000×). Adapted with permission from Kiani et al.^36^ Copyright 2025 Elsevier.

In contrast, the presence of starch improved the mechanical properties of a biomimetic membrane based on PET waste.^35^ Starch particles (0.3, 0.5, and 0.7 wt % %) were deposited by vacuum filtration on a rPET membrane previously obtained by electrospinning to prepare this material. The deposition of starch particles on the rPET was verified by SEM images (Figure 9b). Optimal morphology structure was obtained for the membrane containing 0.5% starch, in which the hydrophilic polysaccharide particles covered the surface of the membrane and there was no agglomeration between them. The tensile strength of the membrane was improved from 1.75 to 4.96 MPa with the addition of 0.5% starch. Additionally, the membrane obtained with 0.5% starch also showed abrasion resistance and chemical stability at pH 3.0, 7.0, and 11.0. FTIR spectroscopy results showed that the interaction between the polymers occurred through hydrogen bonds, and the presence of starch reduced the hydrophobicity of the rPET membrane. Thus, like cellulose, starch reduced the hydrophobicity and improved the mechanical properties of the rPET material. In addition, the incorporation of starch increased the rPET membrane’s abrasion and corrosion resistance.

**Figure 9:**
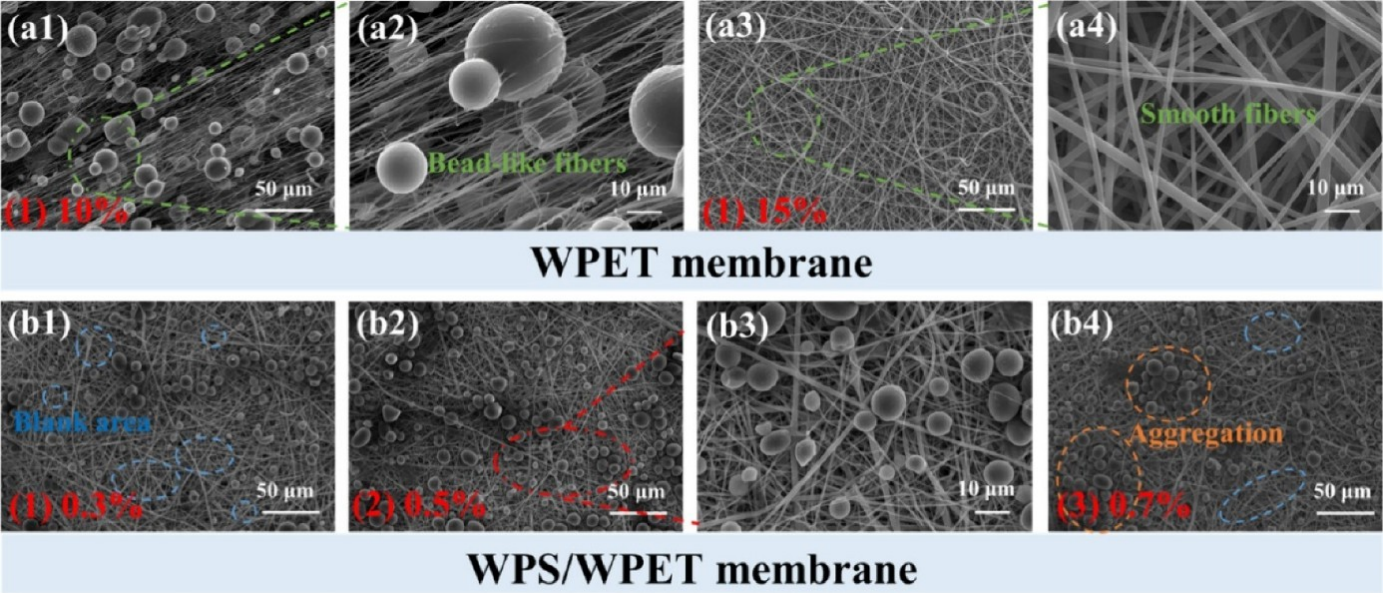
SEM images of different sizes of (a1,a2) beaded rPET fiber membranes and (a3,a4) bead-free rPET fiber membranes, (b1–b4) rPET/starch membranes. Adapted with permission from Xiong et al.^35^ Copyright 2025 American Chemical Society.

### 4.2 Applications of rPET/Polysaccharide Membranes

Membranes based on rPET and polysaccharides have properties that are suitable for applications in a variety of areas. The potential of these materials was reported in water purification^23,32,34–36,39,40^ and mixture separation^38,41^ processes, biocatalysis,^31^ and the biomedical field^27,33^ (Table 1).

The application field depends on the properties and type of material produced. Therefore, this section presents and discusses the applications and performance of the rPET and polysaccharide-based membranes. In addition, new applications are proposed based on the characteristics and properties of rPET/polysaccharide materials.

#### 4.2.1 rPET/Polysaccharide Membranes for Liquid Processes

rPET/polysaccharide membranes have been used mainly in liquid separation processes, including oil–water separation,^23,32,35,39^ drug nanofiltration,^36^ toxic metal removal from aqueous solution,^40^ nanofiltration of organic solvents,^38^ and hydrophilic pervaporation processes,^41^ as presented in Figure 10. The properties and performance of each membrane are summarized in Table 3. The preparation methods used to obtain the membranes were described in the previous section.

**Figure 10:**
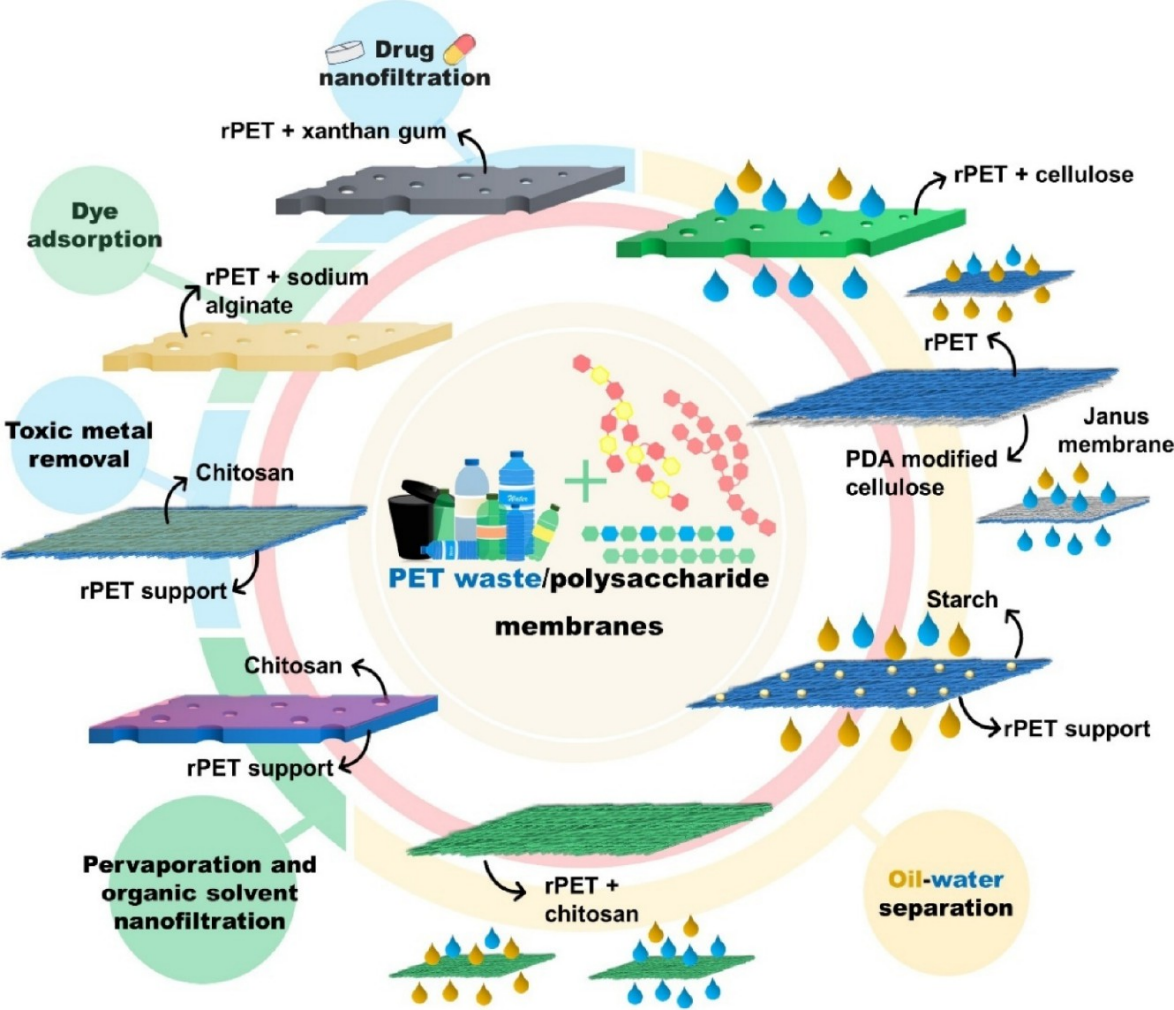
Types of rPET/polysaccharide membranes and applications in liquid processes.

**Table 3:**
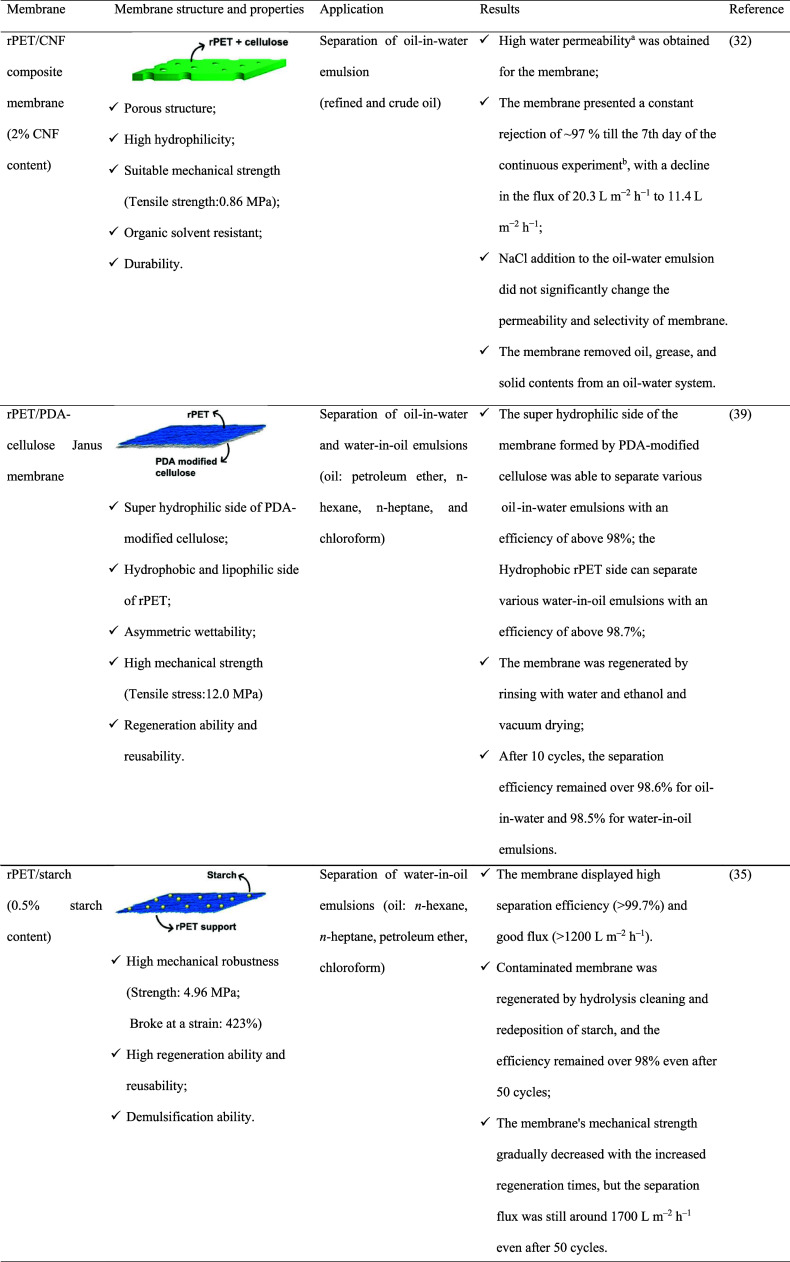

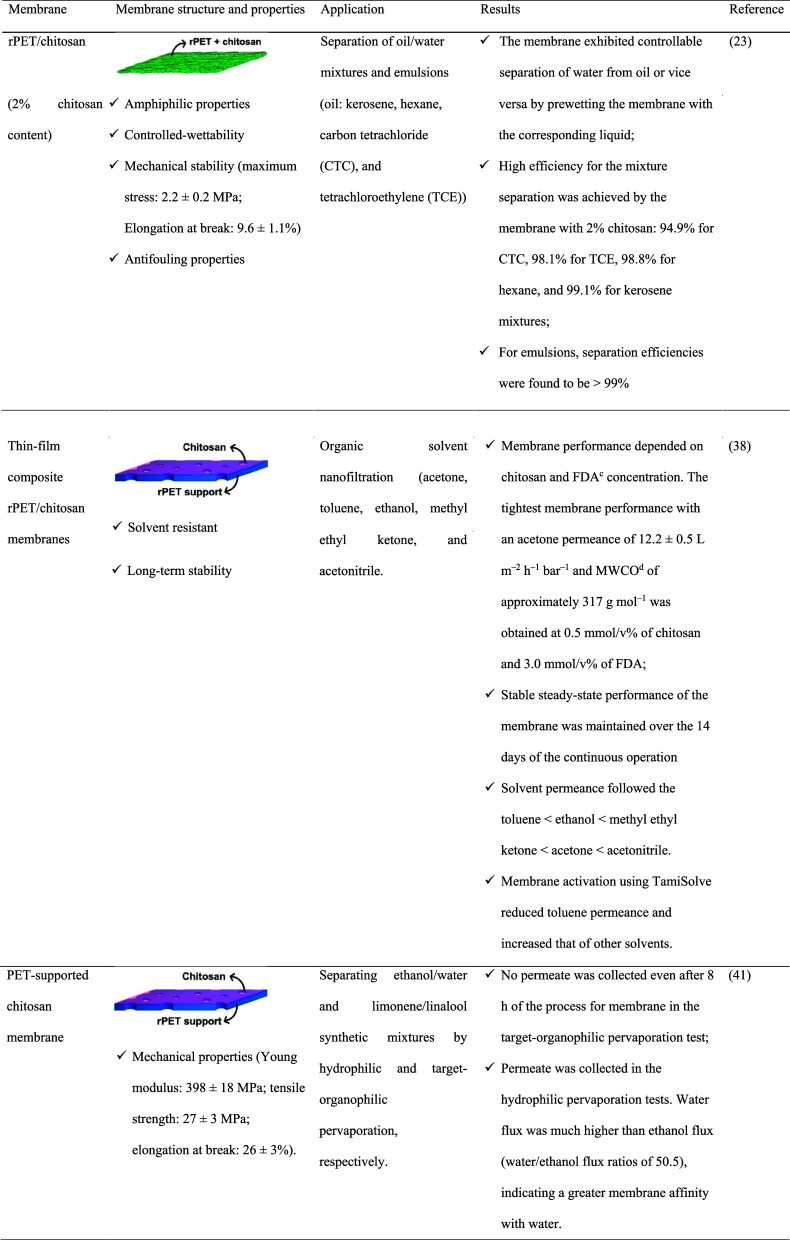

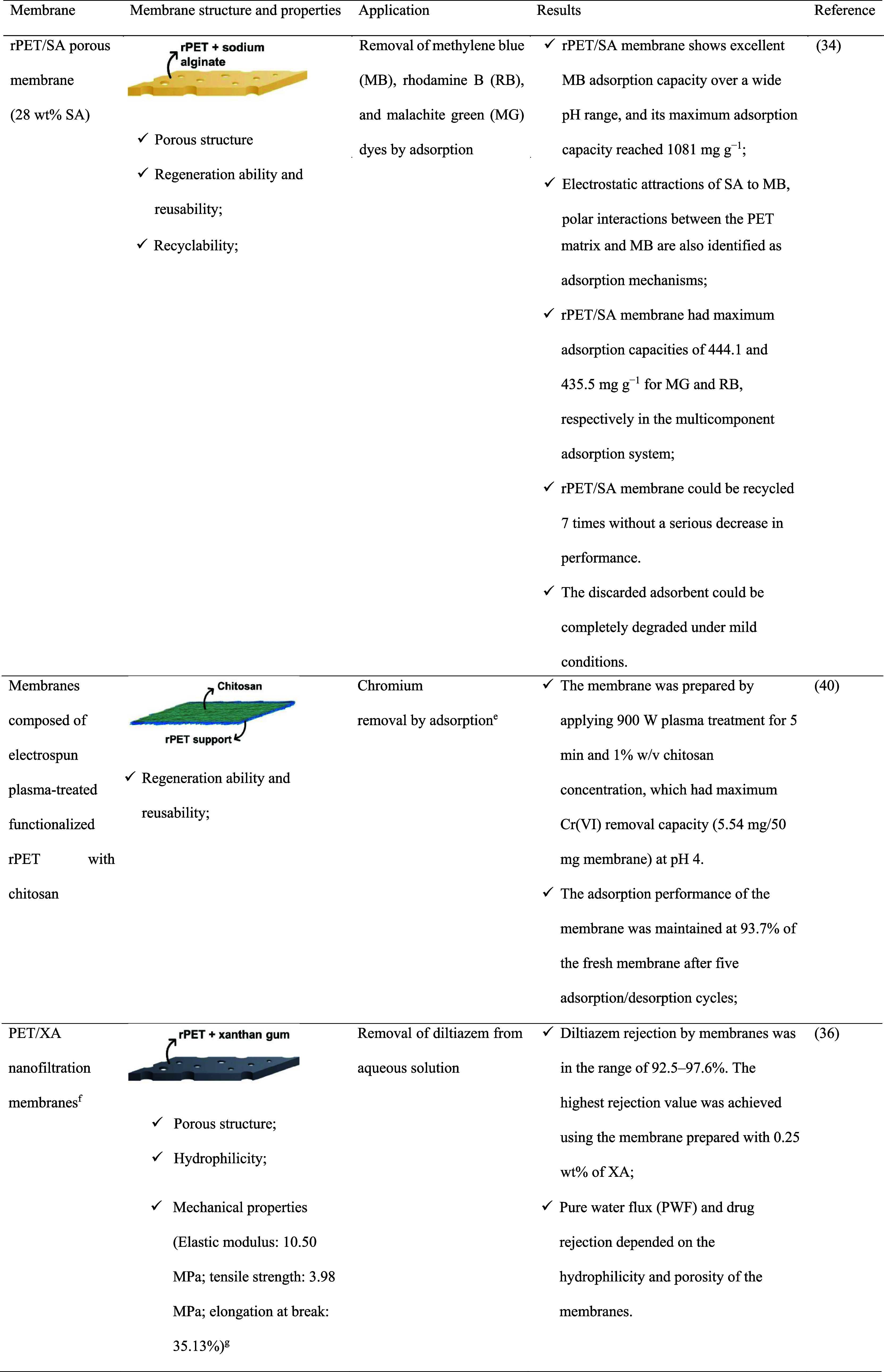
Properties, Applications, and Performance of PET/Polysaccharide Membranes

a In the dead-end and cross-flow permeation experiments.

b Cross-flow permeation experiment.

c 2,5-Furandicarboxaldehyde.

d Defined as the lowest molecular weight solute in which the membrane retains 90% of it.

e A laboratory-scale cross-flow filtration unit performed adsorption experiments.

f Obtained in the methanol coagulation bath.

g For membrane prepared with 0.25 wt % of XA.

Considering the global context, which includes the scarcity of water resources and the increasing pollution of water bodies due to human activities, membrane technologies applied to purifying water have become essential tools in the environmental remediation process.^96^ Polymer materials are the primary choice for membrane fabrication due to their low cost/benefit ratio, high flexibility, and ease of fabrication.^32,97^ However, synthetic polymers, which are generally used to obtain membranes, contribute to greenhouse gas emissions and increased plastic waste.^98^

Using waste PET and polysaccharides to obtain membranes can help reduce the environmental impact of conventional polymeric membranes.^98^ rPET/polysaccharides membranes have hydrophilicity, porosity, and mechanical properties suitable for application in water purification processes.^23,32,39,41^ Hydrophilicity and porosity were important parameters for the separation of oil-in-water emulsion and removal of diltiazem by rPET/cellulose^32^ and rPET/XA^36^ membranes, respectively. With increasing hydrophilicity and porosity, the transport of water molecules through the membrane is also increased.^32,36^ Additionally, increased hydrophilicity improved the rejection efficiency of oil^32^ and drugs^36^ by these membranes. In contrast, neat rPET membranes (without polysaccharides) exhibited inferior water permeability and separation performance due to their lower hydrophilicity compared to rPET/polysaccharide membranes.^32,36^ Improving membrane hydrophilicity is also a strategy to reduce membrane fouling and extend its lifespan.^36^

The inferior performance of the neat rPET membrane was also reported for chromium removal.^40^ However, the presence of chitosan in the membrane led to increased chromium removal at pH 4.0. Under this condition, protonated free amino groups (−NH_3_^+^) of chitosan have the highest potential to interact with the dominant Cr­(VI) species in solution (HCrO_4_^–^).^40^ Therefore, the polysaccharide also introduces new functionalities into the rPET membrane.

Other properties of rPET/polysaccharide membranes include amphiphilicity,^23,39^ asymmetric wettability,^39^ controlled-wettability,^23^ solvent resistance and durability,^32,38^ as well as regeneration ability and reusability.^35,39,40^ These properties were advantageous for the oil–water separation process.^23,32,35,39^ Membranes with amphiphilic properties and asymmetric or controlled wettability could separate water from oil and oil from water.^23,39^ The organic solvent resistance of membranes is significant for treating oil industry wastewater because, in addition to oil, many organic substances are also present.^32^ Furthermore, this property is also desired in membranes for organic solvent nanofiltration.^38^ Finally, regeneration ability, reusability, and durability are essential for increasing the usage time of membranes and reducing environmental pollution.^35^ Therefore, rPET/polysaccharide membranes can be excellent alternatives to conventional polymeric membranes.

#### 4.2.2 Other Applications of rPET/Polysaccharide Membranes

rPET/polysaccharides membranes have also been applied in the biomedical^27,88^ and biocatalysis fields.^31^ The functionalization of rPET membranes with alginate was performed as a strategy to produce a material with antimicrobial properties due to the ability of the polysaccharide carboxylic groups to interact with metal ions.^27^ Initially, the rPET membrane was obtained by electrospinning and subsequently functionalized with alginate and Cu^2+^ ions from a CuCl_2_ solution. rPET/Alginate-Cu^2+^ membranes demonstrated the highest antimicrobial activity against *Staphylococcus aureus*, *Pseudomonas aeruginosa*, and *Candida albicans*, outperforming the control and PET-only samples. In contrast, PET/Alginate-Cu^2+^ samples demonstrated significant reductions in microbial adherence for all three species, while PET-only samples showed high adherence levels for microbial species.^27^

Membranes with antimicrobial properties were also obtained by coating electrospun rPET membranes with chitosan.^88^ Antimicrobial tests demonstrated that rPET/chitosan membranes significantly inhibited *S. aureus*, *P. aeruginosa*, and *C. albicans* biofilm formation compared with control and uncoated rPET surfaces. Therefore, chitosan introduced antimicrobial and antibiofilm properties to the rPET membrane. Given their antimicrobial efficacy and biocompatibility, rPET@CS scaffolds hold promise for biomedical applications such as wound dressings, implant coatings, and infection control. In addition, the biocompatibility of rPET/chitosan membranes was demonstrated. Thus, these materials hold promise for biomedical applications such as wound dressings, implant coatings, and infection control.^88^

In the biocatalysis field, a rPET/cellulose acetate nanofibrous membrane was produced by using the electrospinning method and used as a carrier for porcine pancreatic lipase (PPL).^31^ Cellulose acetate is a soluble esterified derivative of cellulose that is also nontoxic, biocompatible, inexpensive, and biodegradable. PPL was immobilized onto the nanofibers activated with glutaraldehyde. The immobilization process increased the thermal stability of the enzyme (Figure 11). Additionally, the pH stability properties of the PPL enzyme improved after immobilization, especially in the acidic region. Immobilization of the enzyme on the nanofibers also improved the storage capacity. After 12 days of storage, free PPL experienced a significant decrease in activity, retaining only 35% of its initial activity. In contrast, PPL immobilized rPET/CA showed remarkable stability, retaining 89% of its activity during the same period. In addition, immobilized PPL demonstrated significant reusability, retaining more than 50% of its activity after 13 uses. These results demonstrate the potential of the rPET/cellulose acetate nanofibrous membrane for biocatalysis.

**Figure 11:**
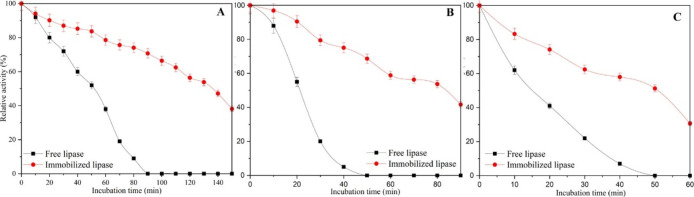
Thermal properties of free lipase and lipase immobilized on rPET/CA nanofiber at (A) 50 °C, (B) 60 °C, and (C) 70 °C. Reprinted with permission from IŞIK.^31^ Copyright 2024 American Chemical Society.

## 5 Recycling and Degradability

Studies on the recyclability and degradability of rPET/polysaccharide materials are still scarce in the literature.^34,89^ Although polysaccharides are biodegradable, their impact on the degradability and recyclability of rPET-based membranes is understood.^34,89^ On the other hand, even materials with excellent reuse performance and durability, like rPET/chitosan,^40^ rPET/PDA-cellulose,^39^ and rPET/starch^35^ membranes, have a limited lifespan. Thus, knowledge about the degradability and recyclability of rPET/polysaccharide membranes is important for waste management, with a focus on sustainability and circularity.

It has been reported that incorporating chitosan into the virgin PET and rPET matrices reduced the decomposition time of the polymer.^89^ Commercial PET and rPET obtained from discarded bottles were extruded with different amounts of chitosan (1, 2.5, and 5 wt %) to form filaments. Their degradation in a real soil environment (6 months) and in accelerated weathering (1200 h) was investigated. Based on the results of accelerated weathering, the lifetime of the materials was estimated. The incorporation of 5% chitosan into the PET and rPET matrices reduced the lifetime from 125 to 60 years, and from 86 to 45 years, respectively. The highest weight loss of polymer blends was observed in the rPET matrix, which was associated with a loss of properties due to reprocessing. Degradation in soil was also favored when higher amounts of chitosan (5%) were added to PET matrices.^89^ Therefore, the presence of chitosan improved the degradability of both PET matrices. In contrast, Aldas et al.^99^ found that no significant degradation of PET was obtained due to the presence of thermoplastic starch (TPS) after 8, 21, and 30 days of incubation under composting conditions.

On the other hand, recycling rPET/polysaccharide materials is a challenge. Although PET is recyclable, the introduction of other polymers can negatively impact its reprocessing.^21,100^ The effect of small quantities of TPS on the mechanical recycling of rPET was investigated.^99^ The TPS (0, 2.5, 5, 7.5, 10, and 15 wt %) was melt-blended with rPET in a twin-screw extruder. It was found that the mechanical properties (tensile strength, elongation at break, and Young′s modulus) of rPET decrease with the presence of TPS.^99^ Indeed, the mechanical recycling method is limited to single polymer waste.^101^

However, some studies show that chemical recycling may be suitable,^34,101^ although most methods require high energy, high cost, intense reaction conditions, and specialized equipment.^18,19^ Yu et al.^34^ observed that the fresh and spent (after 7 dye adsorption–desorption cycles) SA@rPET membrane could be degraded by using the mild alkaline hydrolysis method. This chemical recycling consists of the depolymerization of the polymer in an aqueous medium, which can result in the formation of TPA and EG.^93,102^ The rPET matrix of the SA@rPET composites was depolymerized to TPA solids. At the same time, SA was retained in the degraded solution (Figure 12). The FT-IR, ^1^H NMR, and ^13^C NMR results showed that TPA obtained by degradation of both fresh and spent SA@rPET membranes had a structure and purity identical to that of commercial TPA.^34^ Therefore, the incorporation of a biopolymer did not hinder the chemical recycling of PET. TPA can be used for the repolymerization of PET or other applications, which facilitates the circularity of the polymer.

**Figure 12:**
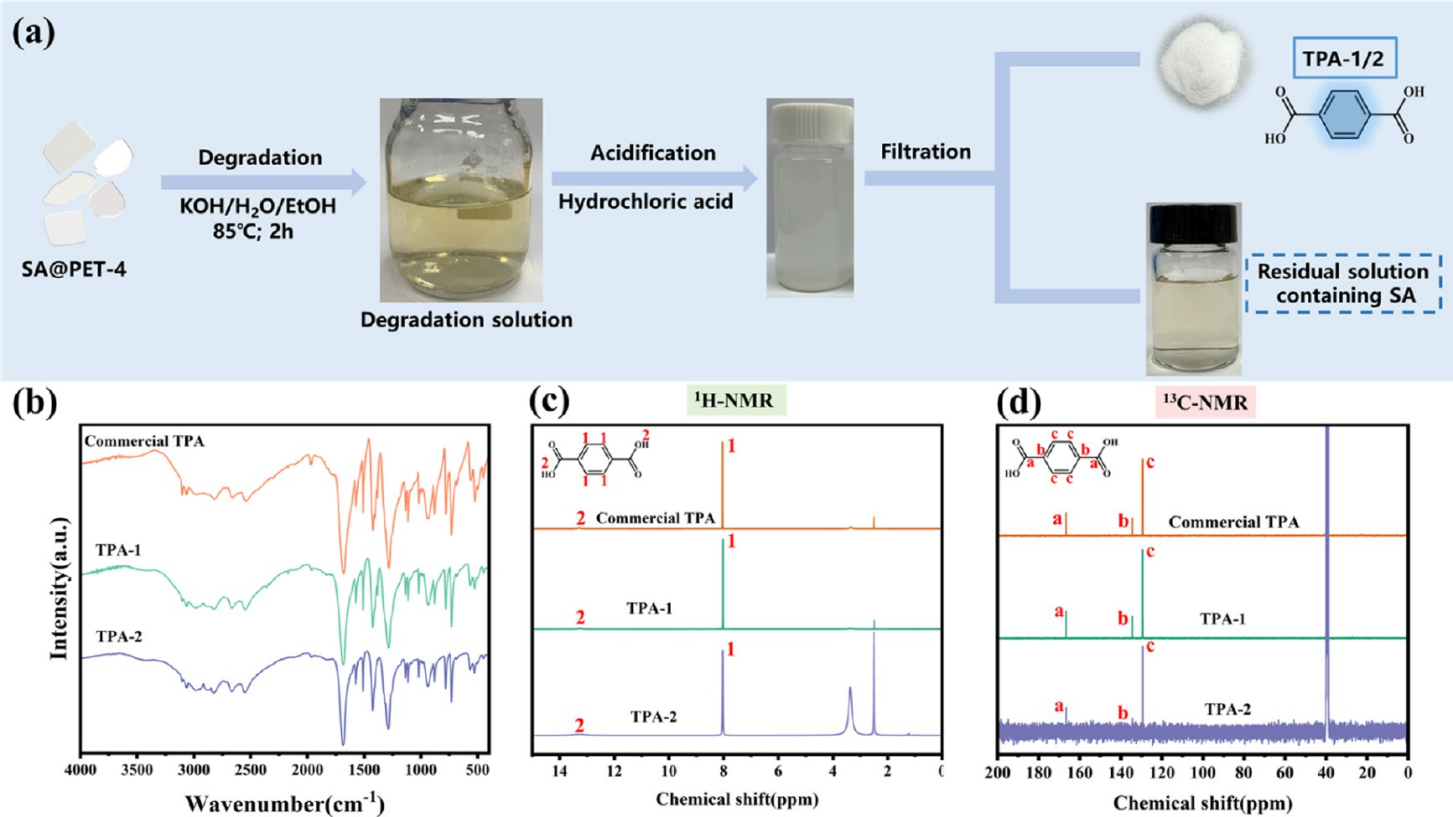
(a) Flowchart of adsorbent degradation; (b) FTIR; (c) H^1^ NMR; and (d) C^13^ NMR spectra of commercial TPA, TPA-1 (obtained by degradation of fresh SA@PET), and TPA-2 (obtained by degradation of SA@PET after 7 cycles of recycling). Reprinted with permission from Yu et al.^34^ Copyright 2024 Elsevier.

Chemical recycling is also used for polycotton (cellulose plus poly­(ethylene terephthalate)) recycling.^101^ The strategies investigated were discussed in a previous review^101^ and include (i) preferential dissolution of PET using a suitable solvent; (ii) depolymerization of one polycotton component using acidic, alkaline, or enzymatic catalysts, leaving the other less affected by the treatment; or (iii) dissolution of cellulose in a suitable solvent, followed by PET/solution separation and regeneration of the dissolved biopolymer using a suitable nonsolvent. These strategies can be helpful for the recycling of rPET/cellulose materials.

## 6 Industrialization Opportunities and Challenges

rPET/polysaccharide membranes have demonstrated potential for application in fields related to environmental protection,^23,32,34–36,39,40^ biomedicine,^27,33^ and biocatalysis,^31^ among others. In addition, environmental pollution caused by PET waste has driven demand for rPET products.^103^ The market size of rPET-based materials is expected to grow significantly in the coming years due to the demand for advanced materials with enhanced properties and sustainability features.^103^

However, the processes for obtaining rPET/polysaccharide membranes are still limited to a laboratory scale. The transition from laboratory-scale production to large-scale manufacturing presents some challenges.^103^ Electrospinning is the most widely used technique for obtaining membranes based on rPET and polysaccharides.^23,27,33,35,37,39,40,64,65,88^ Indeed, it was found that the electrospun membranes exhibited superior mechanical performance^35,37,39,64^ than those obtained using the NIPS technique.^32,36^ However, although electrospinning is a technology with low energy consumption, its industrial scale-up poses significant challenges due to technical and operational limitations, particularly in achieving consistent quality and high production rates.^104,105^

For industrialization, the cost and quality of the raw materials must also be considered. The use of postconsumer PET bottles or mechanically recycled PET in the preparation of materials combined with polysaccharides has some advantages and disadvantages that must be considered. rPET is more cost-effective than virgin PET. Energy required for the production of virgin PET (78–86 MJ/kg) is greater than that for recycling it (27–30 MJ/kg).^44^ However, rPET obtained by mechanical recycling usually has inferior properties to virgin PET^26^ because the extrusion process of the polymer can result in degradation of polymer chains, which can directly affect the mechanical performance of the product.^106–108^ However, studies have shown that mechanically recycled PET/CNCs^37^ and virgin PET/CNCs^109^ had similar dynamic-mechanical (storage modulus, glass transition temperature) and tensile (tensile strength) properties. In both studies, the membranes were fabricated using 10 wt % of CNCs and 2.5 wt % castor oil by electrospinning.^37,109^

On the other hand, direct recycling of PET bottles eliminates prior recycling steps (mechanical, chemical, or other), which reduces production costs. However, factors such as lifespan, production batch, presence of contaminants, source, and type of PET bottles must also be carefully considered for the quality and reproducibility of the final product. In fact, ensuring uniform product quality is a crucial aspect of scale-up.^103^ For instance, postconsumer PET water or soft-drink bottles used for the preparation of rPET/polysaccharide materials have different characteristics.

PET water bottles (water grade) have low intrinsic viscosity and acetaldehyde suppression and could have additives to enable ultrathin bottle walls.^108^ In contrast, PET soft-drink bottles (carbonated soft drink grade) have the highest intrinsic viscosity and comonomers to resist expansion.^108^ In addition, PET soft-drink bottles can be light-colored or dark-colored, while PET water bottles (water grade) are light-colored.^110^ These factors must also be considered if mechanically recycled PET is used because different types of PET bottles were used; the final rPET pellet may have many different comonomers, additives, and levels of additives.^108^ So, proper disposal, collection, and sorting of postconsumer PET bottles are essential to obtain rPET/polysaccharide membranes.

However, the collection strategies of waste PET vary in developed and developing countries.^5,21^ Developed countries employ curbside and deposit systems, while informal waste pickers dominate in developing countries.^21^ In addition, the absence of adequate infrastructure in developing countries often leads to inefficient sorting, low recovery rates, and inadequate treatment of PET waste.^21^

Therefore, there are many challenges for the industrialization of rPET/polysaccharide membranes, including the large-scale production of rPET-based materials, identification of dependable suppliers, establishment of a consistent supply chain for raw materials, and the requirement of quality control and characterization measures.^103^

## 7 Prospect

This review describes recent strategies for PET recycling that combine postconsumer PET with polysaccharides to obtain membranes. Despite the potential and performance of these materials in different applications, efforts are required to fill some knowledge gaps identified in the studies. Knowledge gaps are mainly related to understanding (i) the effect of the PET source on membrane performance and the influence of polysaccharides on (ii) degradability and (iii) recyclability of rPET/polysaccharide membranes. Therefore, future research should focus on:

i. Evaluate the effect of using different PET sources (PET bottle waste, mechanically and chemically recycled PET, and virgin PET) in the preparation of membranes combined with polysaccharides. This study is important for comparing the properties of the membranes and verifying the practical feasibility of PET upcycling into rPET/polysaccharide membranes.
ii. Investigate the reproducibility of rPET/polysaccharide membranes and standardize preparation methods.
iii. Propose economical and efficient methods for recycling rPET/polysaccharide membranes, and investigate their degradability considering the effect of different types of polysaccharides used. Recyclability and degradability are important for the circular economy and sustainability of rPET-based materials.^5^
iv. Evaluate other polysaccharides in the preparation of rPET-based membranes.
v. Explore the synergistic potential of different polysaccharide combinations. So far, studies have focused on the combination of rPET with single polysaccharides, but composite polysaccharides may produce better effects.
vi. Evaluate the potential of rPET/polysaccharide membranes in gas separation processes. Membranes are used to separate nitrogen from air, capture CO_2_, and purify hydrogen. The association with other polymers with functional groups (e.g., −OH, −NH_2_, and −COOH) that facilitate interaction with the molecule to be retained.^111,112^ For instance, the amino groups of chitosan are capable of interacting with CO_2_.^113^ Thus, rPET/chitosan membranes may be useful for CO_2_ capture.

## 8 Conclusions

rPET and polysaccharides can be combined using different synthesis strategies to obtain membranes with properties absent from the isolated polymers. Polysaccharides can introduce or improve the properties of rPET-based materials and insert new functionalities, depending on the synthesis method used. Electrospinning has proven to be a promising method for obtaining membranes with a high mechanical performance. The NIPS technique has been used to produce porous rPET/polysaccharide membranes. The porosity and hydrophilicity of the membranes can be controlled by varying the polysaccharide content. However, this method produces membranes with a limited number of mechanical properties.

The polysaccharide type also influences the properties of the membranes. Cellulose and starch improved the mechanical properties and hydrophilicity of the rPET membranes obtained by electrospinning. These rPET/cellulose and rPET/starch membranes showed excellent regeneration and reuse capacity in oil–water separation processes. On the other hand, chitosan, alginate, and xanthan gum were primarily used to introduce new functional groups into the rPET matrix and to modulate its wettability. Membranes containing these polysaccharides could be used to remove pollutants, such as toxic metals, dyes, and drugs. Although rPET/polysaccharide materials have been obtained mainly for water purification processes, this upcycling strategy can potentially increase the application fields of the recycled polymer. In fact, the potential of these materials in mixture separation processes and biomedical and catalytic applications has also been demonstrated. However, new strategies must be proposed to overcome the challenges related to the source of PET, the degradability, and the recyclability of rPET/polysaccharide membranes.

## Supplementary Material



## Notes

SEM images of the neat PET matrix obtained by the NIPS method with different TFA/DCM mass ratios and different PET mass fractions; TGA and DTG curves of PET, SA, and SA@PET samples; thermal analysis results of PET, SA, and SA@PET samples; specific surface area and porosity of the SA@PET absorbents; elemental content of SA@PET-4 after MB adsorption; adsorption isotherms of SA@PET-4 for MG and RB; Langmuir, Freundlich, and Redlich–Peterson parameters for the adsorption of MG and RB on SA@PET-4; UV absorption spectra; reusability of SA@PET-4; physicochemical characteristics of MB, RB, and MG; SEM images of SA@PET-4 adsorbents prepared with recycled TFA/DCM solvent; FTIR spectra and adsorption capacity of the SA@PET-4 adsorbent prepared by the recycled TFA/DCM solvent; flow chart of adsorbent degradation; and FTIR, H^1^ NMR, and C^13^ NMR spectra of commercial TPA and the TPA obtained by SA@PET-4 degradation (PDF)
