# Supplementary material for: PET Waste Upcycling with Polysaccharides: Promising Alternative for Sustainability—Review
Source: ACS Omega. 2025 Dec 18;11(7):10899–920. doi: 10.1021/acsomega.5c06399 (PMC12946979; doi:10.1021/acsomega.5c06399)
Supplement: Supplementary file 1 [file ao5c06399_si_001.pdf]

Supplemental Material

Waste Treats Waste: Facile Fabrication of Porous Adsorbents from Recycled PET and Sodium Alginate for Efficient Dye Removal

Jing Yu, Lan Bai*, Zijun Feng, Lin Chen, Shimei Xu, Yuzhong Wang*

Collaborative Innovation Center for Eco-Friendly and Fire-Safety Polymeric Materials (MoE), State Key Laboratory of Polymer Materials Engineering, National Engineering Laboratory of Eco-Friendly Polymeric Materials (Sichuan), College of Chemistry, Sichuan University, Chengdu 610064, China.

**E-mail: bailanchem@scu.edu.cn; yzwang@scu.edu.cn*

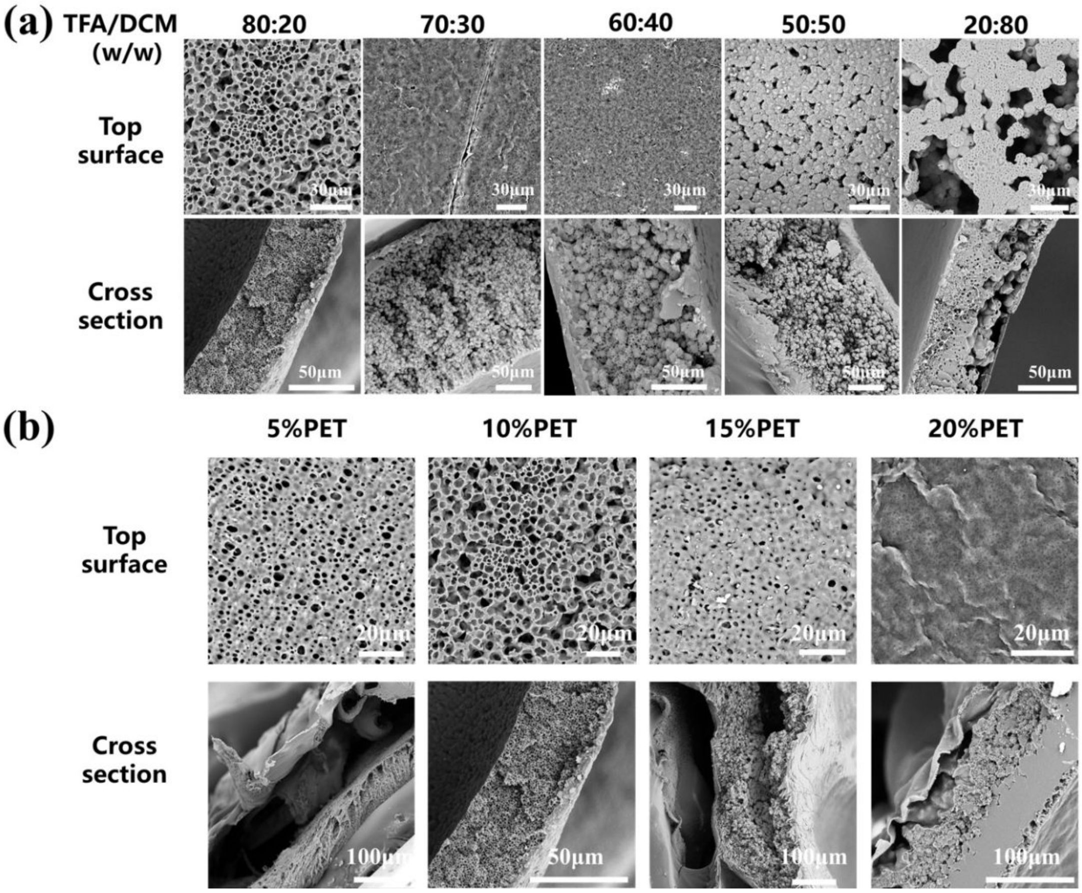

Fig. S1 SEM images of the neat PET matrix obtained by the NIPS method with (a) different TFA/DCM mass ratios and (b) different PET mass fractions.

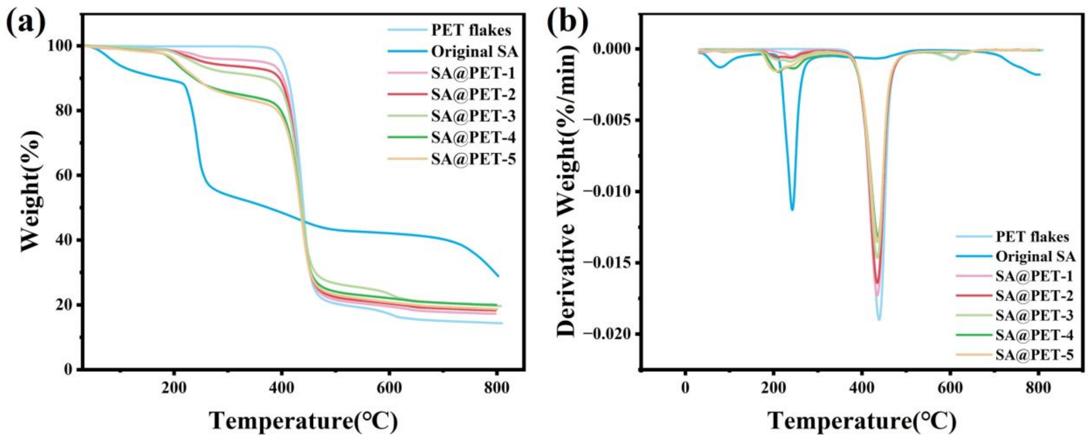

Fig. S2 (a) TGA and (b) DTG curves of PET, SA and SA@PET samples.

Table S1 Thermal analysis results of PET, SA and SA@PET samples.

Sample	$T_{5\%}$ (℃)	T_{max} (℃)	Residue (%)
PET flakes	402.1	440.1	14.3
Original SA	89.4	223.0	28.8
SA@PET-1	364.9	435.4	17.3
SA@PET-2	256.2	435.7	18.3
SA@PET-3	236.9	436.1	20.5
SA@PET-4	205.6	436.4	20.7
SA@PET-5	201.4	436.6	20.0

Table S2 Specific surface area and porosity of the SA@PET absorbents.

Samples	Specific surface area (m^2/g)	Porosity (%)
Neat PET matrix	11.42	45.9
SA@PET-1	23.59	55.3
SA@PET-2	28.78	61.3
SA@PET-3	31.53	60.7
SA@PET-4	23.06	60.7
SA@PET-5	20.97	64.2

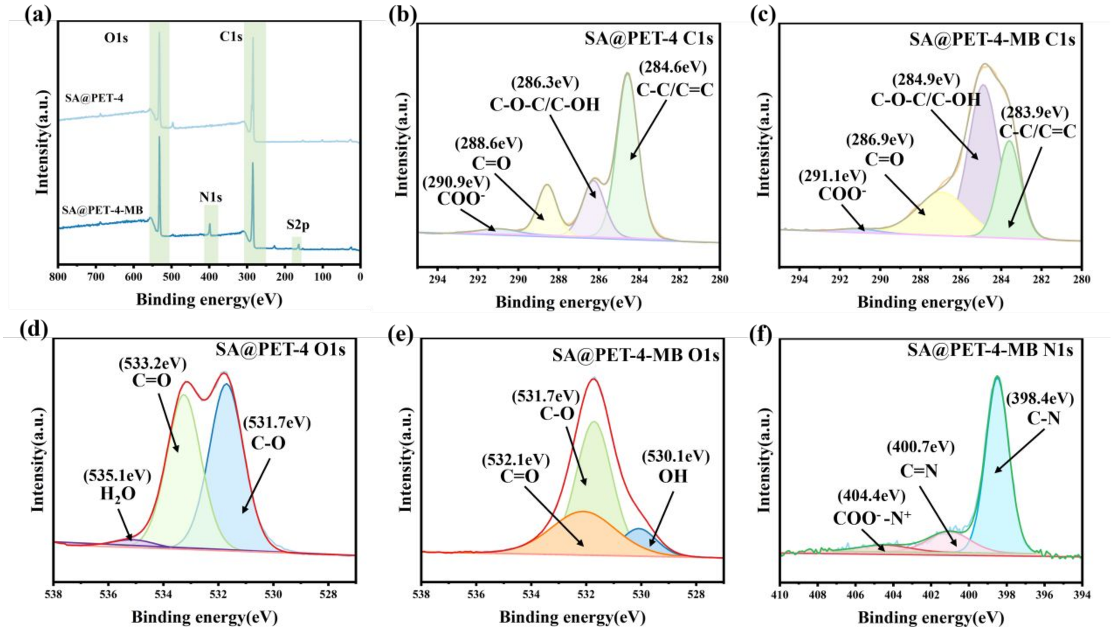

Fig. S3 (a) Wide-scan XPS spectra of SA@PET-4 and SA@PET-4-MB; high-resolution core-level C 1s spectra of (b) SA@PET-4 and (c) SA@PET-MB; high-resolution core-level O 1s spectra of (d) SA@PET-4 and (e) SA@PET-4-MB; (f) high-resolution core-level N 1s spectrum of SA@PET-4-MB.

Table S3 Elemental content of SA@PET-4 after MB adsorption

Element (%)	Top surface	Cross section
C	57.81	52.92
O	36.05	38.94
N	6.00	8.14
S	0.14	0.00
Cl	0.00	0.00

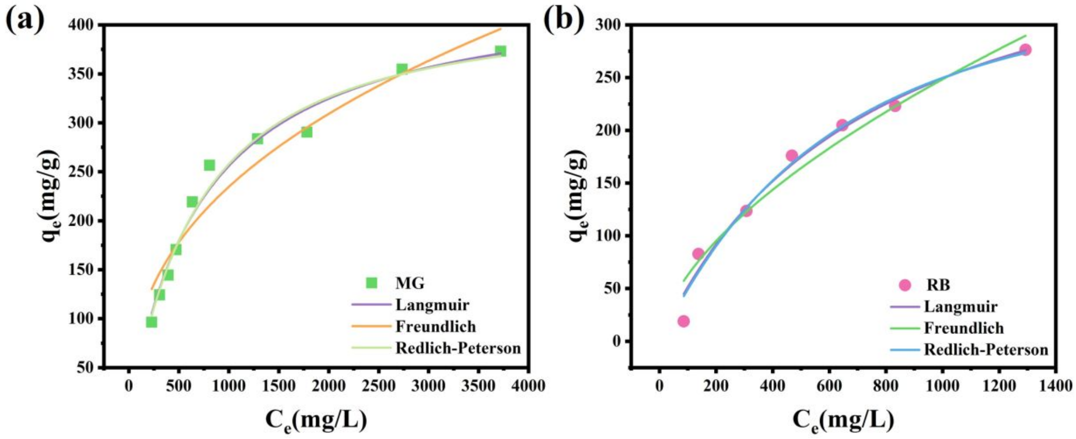

Fig. S4 Adsorption isotherms of SA@PET-4 for (a) MG and (b) RB.

Table S4 Langmuir, Freundlich and Redlich–Peterson parameters for the adsorption of MG and RB on SA@PET-4.

	Langmuir model			Freundlich model			Redlich-Peterson model			
	Q_m (mg g^{-1})	K_L (L/mg)	R^2	n	K_F (L/mg)	R^2	a (L/mg)	K_{RP} (L/g)	β	R^2
MG	444.1	0.0014	0.9809	2.5103	14.9697	0.9360	9.31×10^{-4}	0.5726	1.0394	0.9812
RB	435.5	0.0013	0.9708	1.6727	3.9980	0.9529	4.03×10^{-4}	0.5306	1.1484	0.9787

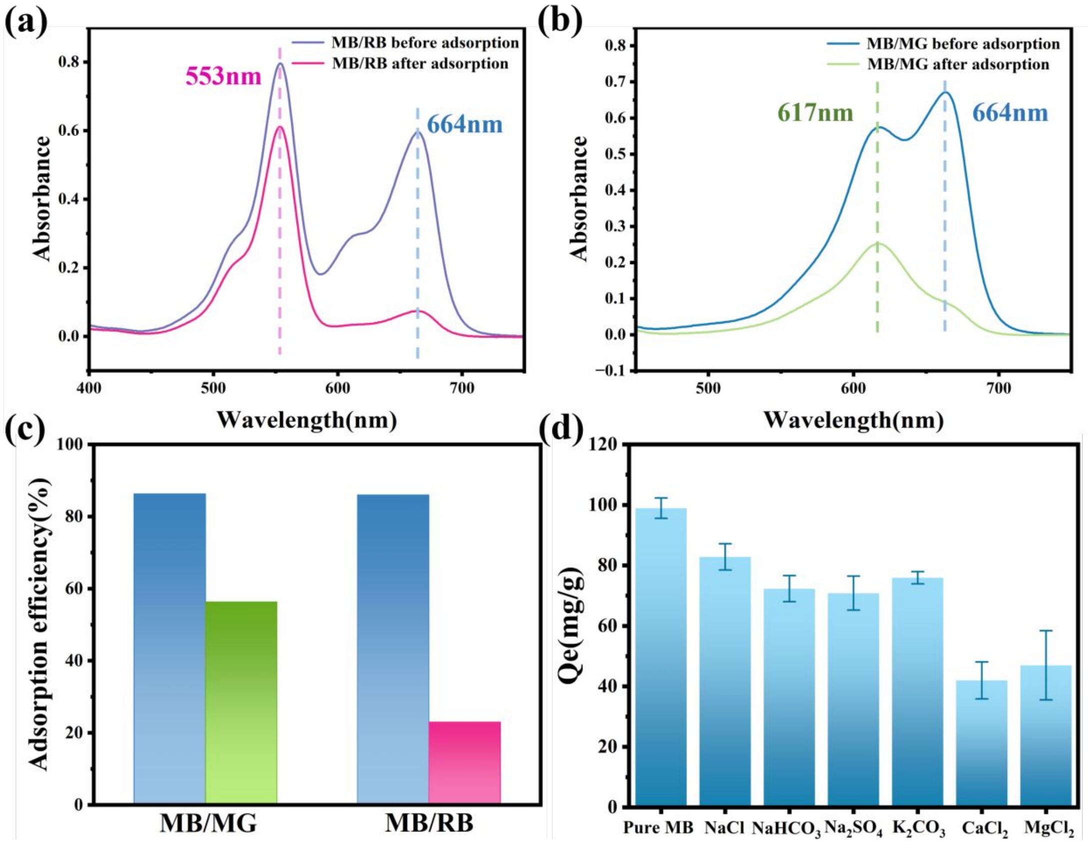

Fig. S5 UV absorption spectra of (a) MB-RB and (b) MB-MG binary solutions before and after adsorption; (c) adsorption efficiency of dyes in the binary solution; (d) ion interference on the adsorption capacity of SA@PET-4 for MB.

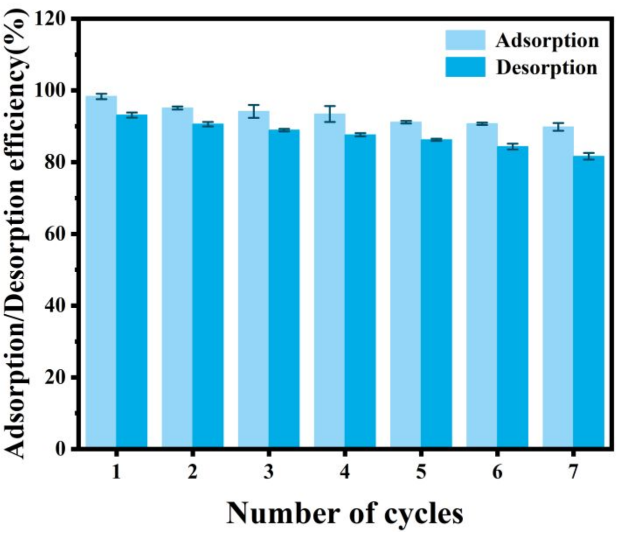

Fig. S6 Reusability of SA@PET-4.

Table S5 Physicochemical characteristics of MB, RB and MG.

Dye	Abbreviation	M.W. [a]	λ_{max}[b] (nm)	Molecular structure	Category
Methylene Blue	MB	319.85	664	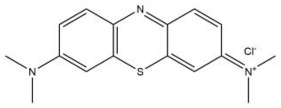	cationic
Rhodamine B	RB	479.01	553	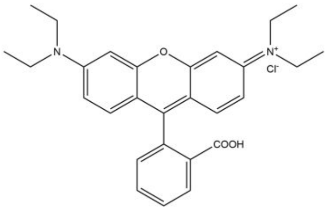	cationic
Malachite green	MG	364.91	617	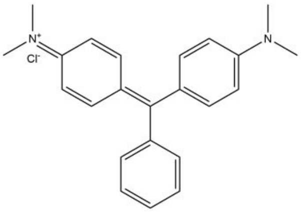	cationic

[a] Molecular weight (g/mol)

[b] maximum absorption wavelength (nm)

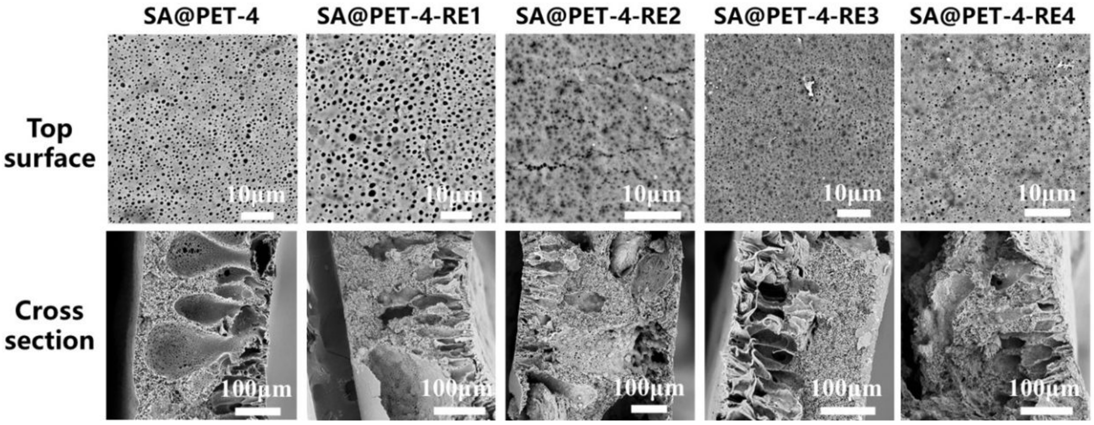

Fig. S7 SEM images of SA@PET-4 adsorbents prepared with recycled TFA/DCM solvent.

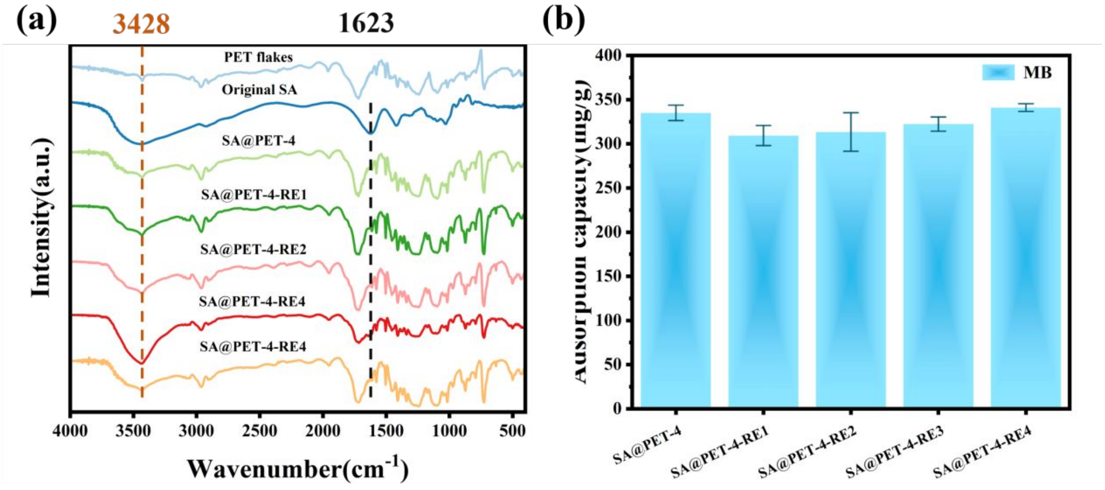

Fig. S8 (a) FTIR spectra and (b) adsorption capacity of the SA@PET-4 adsorbent prepared by the recycled TFA/DCM solvent.

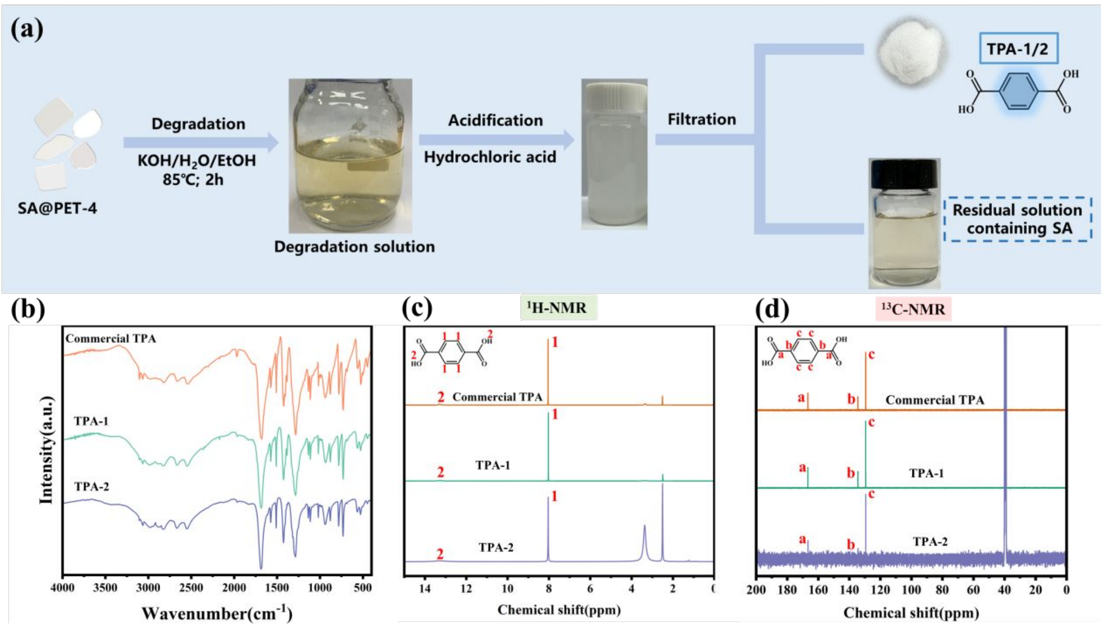

Fig. S9 (a) Flow chart of adsorbent degradation; (b) FTIR; (c) H^1 NMR; and (d) C^{13} NMR spectra of commercial TPA and the TPA obtained by SA@PET-4 degradation and.
